# Taxonomic implications of normal and abnormal stomatal complexes in *Indigofera* L. (Indigofereae, Faboideae, Fabaceae)

**DOI:** 10.1007/s00709-024-01951-0

**Published:** 2024-04-19

**Authors:** Mohamed O. Badry, Ahmed K. Osman, Mostafa Aboulela, Shereen Gafar, Iman H. Nour

**Affiliations:** 1 Department of Botany & Microbiology, Faculty of Science, South Valley University, Qena, 83523 Egypt; 2 Botany and Microbiology Department, Faculty of Science, Assiut University, Assiut, 71516 Egypt; 3 Botany and Microbiology Department, Faculty of Science, Alexandria University, Alexandria, 21511 Egypt

**Keywords:** Abnormal stomata, Egypt, Foliar epidermis, Giant stomata, *Indigofera*, Taxonomic implications

## Abstract

This study is the first to report the foliar and stem epidermal micro-morphology of 13 taxa of *Indigofera* L. (Fabaceae) using light (LM) and scanning electron microscopy (SEM). The micro-morphological characteristics studied here are related to the epidermal cell shape, size, frequency, anticlinal wall pattern, and stomatal complex types, size, position, frequency, and index. The study revealed 19 major normal stomatal types with eight subtypes and seven major abnormal stomatal types with 13 subtypes. The stomatal index was lower on the abaxial leaf surface than on the adaxial surface. Notably, the adaxial surface of *I. hochstetteri* had the highest stomatal index (27.46%), while the abaxial surface of *I. oblongifolia* had the lowest (9.95%). The adaxial surface of *I. hochstetteri* also displayed the highest average stomatal frequency (38.67), while the adaxial surface of *I. spinosa* had the lowest average frequency (9.37). SEM analysis revealed that most leaves had slightly sunken to sunken stomata, while stem stomata were positioned at the same level as epidermal cells in most taxa. Indigofera's foliar and stem epidermal anatomy recommends their application as baseline data coupled with other taxonomic data for the delimitation and differentiation of closely related taxa in the genus. The study provides a comprehensive description, illustrations, images, and micrographs of the stomatal types, as well as a taxonomic key for distinguishing the studied taxa of *Indigofera*.

## Introduction

*Indigofera* L. is a well-defined genus of the tribe Indigofereae, sub-family Faboideae of the Fabaceae. It is the third largest genus after *Astragalus* L. and *Acacia* Mill (s.l.), comprising about 713 accepted species (POWO 2024). The genus is distributed throughout the world's tropical, subtropical, and arid areas. However, the predominant centres of diversity are Africa and Madagascar (550 species), the Sino-Himalayan area (150 species), Australia (50 species), and the New World (45 species) (Schrire et al. 2009; Chauhan and Pandey 2014). Besides the ecological and medicinal importance of the genus, its members are used as livestock feed, ornamental plants, food coloring additives, and indigo dye products (such as *I. tinctoria* L. and *I. suffruticosa* Mill.) (Marquiafável et al. 2009; Schrire 2013; Gerometta et al. 2020; Shadordizadeh et al. 2023).

Most species of *Indigofera* are shrubs, and some are small bushes, herbaceous perennials, or annuals (Liu et al. 2022). Linnaeus described the genus in 1753 based on the three species: *I. tinctoria*, *I. hirsuta,* and *I. glabra* L. Later, *I. tinctoria* was selected as the lectotype of the genus (Fawcett and Rendle 1920; Mattapha and Chantaranothai 2012).

Comparative anatomical, phylogenetic, and paleo-botanical studies have established the significance of stomatal characteristics and ontogeny as taxonomic criteria at different levels of the systematic hierarchy (Metcalfe and Chalk 1950, 1979; Stace 1965a; Cutler 1969; Fryns-Claessens and Van Cotthem 1973; Dilcher 1974; Dehgan 1980; Gill and Nyawuame 1991; Khan et al. 2013; Zhao et al. 2022). Moreover, some plants are characterized by a specific type of epidermal features, which were shown to be of systematic value (e.g., cuticular characteristics, epidermal cells, stomata, and subsidiary cells) (Redford 1974; Park 1994; Badry 2018; Elkordy et al. 2022; Varilla González et al. 2023).

Leaf epidermal characteristics have been helpful tools in taxonomical revisions of some genera of the Fabaceae. However, few studies have evaluated the detailed comparative micro–morphological characteristics of foliar and stem epidermis and their systematic value within the genus *Indigofera* either as part of studies at the family level or in the description of certain species (Solereder 1908; Metcalfe and Chalk 1950; Vijay-Kumar and Ramayya 1987; Quesada 1997).

In dicotyledonous plants, stomata are developed through a series of specialized cell lineage, including multiple asymmetric divisions and one symmetric division. Dramatic changes in morphology mark each transitional state of stomatal development (Le et al. 2014). The stomatal lineage divisions lead to the formation of different types of cells, including meristemoid mother cells, meristemoids (small triangular cells), stomatal-lineage ground cells (larger cells), guard mother cells (oval-shaped), and finally, guard cells(Bergmann and Sack 2007; Pillitteri and Dong 2013). Initially, the meristemoid mother cell divides asymmetrically, resulting in a small meristemoid and a large stomatal lineage ground cell. The meristemoid cell undergoes up to three or four successive asymmetric divisions and then differentiates into a guard mother cell, which finally undergoes a single symmetric division to form a pair of guard cells (Pillitteri and Dong 2013; Aboulela et al. 2017; Rudall 2023).

In Egypt, Täckholm (1956) reported nine *Indigofera* species. Later, the species number increased to 14 (Täckholm 1974; Boulos 1995; El Hadidi and Fayed 1995). However, only 13 taxa of *Indigofera* were recently listed in the Egyptian flora after removing *I. tritoides* Baker from Täckholm's list (Boulos 1999, 2009; Hosni 2000).

The present study aims to describe the stomatal complex diversity in 12 species and one variety of the genus *Indigofera*, focusing on the micro-morphology of epidermal pavement cells, the stomatal types, frequency, distribution, and the stomatal indices with hypotheses about the associations among these features.

## Materials and methods

### Plant materials

Mature leaves and stems were taken from herbarium specimens of the 13 *Indigofera* taxa kept in the herbaria of Aswan University (ASW), South Valley University, Assiut University (ASTU), and Cairo University (CAI) in Egypt (herbarium acronyms follow Thiers 2023) (Table 1). Plant taxa were identified according to the previously published work (Täckholm 1974; Boulos 1999). The taxonomic names and their authorities were revised according to Plants Of the World Online (POWO 2024), published by the Royal Botanic Gardens, Kew.

**Table 1:** List of the studied *Indigofera* taxa and their locations and collection dates, collectors, and vouchers

NO	Taxon	Locality	Geo–region date	Collector	Voucher
1	***Indigofera arabica* Jaub. & Spach**	Sinai, Egypt	25/3/2006	A. K. Osman	Egypt: S.V.U herbarium
2	***Indigofera argentea* Burm.f**	Wadi um Saadat, Red Sea wadies, Egypt	11/3/1988	M. G. Sheded & M. A. Badri	Egypt: ASW herbarium
3	***Indigofera articulata* Gouan**	Red Sea wadies, Egypt	17–20/1/1987	I. Springuel, M. Badri & M. Salah	Egypt: ASW herbarium Egypt: S.V.U herbarium
4	***Indigofera coerulea* var*. coerulea* Roxb**	Umm Al – Sabaya Island: 50 km SW of Al Qunfida town, about 13 km from seashore, Saudi Arabia	14/2/2001	A. Fayed	Egypt: ASTU herbarium Egypt: S.V.U herbarium
5	***Indigofera colutea* (Burm.f.) Merr**	Halaib Triangle area, Gebel Hamra Dom, Egypt	29/1/2019	T. Ramadan, A. Faried, A. Amro & M. Aboulela	Egypt: ASTU herbarium
6	***Indigofera cordifolia* B. Heyne ex Roth**	Jabel Geili Butava, Sudan	28/9/1967	Kavar–obeid B.25	Egypt: CAI herbarium
7	***Indigofera hochstetteri* Baker**	Halaib Triangle area, Gebel Elba, wadi yahmeeb, Egypt	29/9/2019	T. Ramadan, A. Faried, A. Amro & M. Aboulela	Egypt: ASTU herbarium Egypt: S.V.U herbarium
8	***Indigofera oblongifolia* Forssk**	Nile valley: Abu–Subeira, Egypt	1/11/1984	A. Shaheen	Egypt: ASW herbarium Egypt: S.V.U herbarium
9	***Indigofera sessiliflora* DC**	Gebel Elba, Wadi Yahmeeb, Egypt	25/3/2020	A. K. Osman	Egypt: S.V.U herbarium
10	***Indigofera spiniflora* Hochst. ex Boiss**	Gebel Elba, Wadi Laseitit, Egypt	25/3/2020	A. K. Osman	Egypt: S.V.U herbarium
11	***Indigofera spinosa* Forssk**	Halaib Triangle area, Gebel Elba, wadi Akaw, Egypt	30/1/2019	T. Ramadan, A. Faried, A. Amro & M. Aboulela	Egypt: ASTU herbarium Egypt: S.V.U herbarium
12	***Indigofera subulata *Vahl ex Poir. var. *subulata***	Halaib Triangle area, Gebel Elba, wadi Kansesrob, Egypt	29/1/2019	T. Ramadan, A. Faried, A. Amro & M. Aboulela	Egypt: ASTU herbarium
13	***Indigofera trita* L. F**	Gebel Elba, Wadi Yahmeeb, Egypt	25/3/2020	A. K. Osman	Egypt: S.V.U herbarium

### Epidermal peeling and observation

Epidermal peels were taken from chosen parts of stems and leaves. The leaves were taken from the mid-lamina position at the second node below the apical bud, which is considered the least variable (Metcalfe and Chalk 1979). The samples were treated with 88% lactic acid in a water bath for 5–10 min at 100°C to clear the cuticle and soften the tissue (Badry et al. 2019). Slides of epidermal peels of the stem, both the adaxial and abaxial epidermal layers of the leaf, were made, then mounted in clean 88% lactic acid (Clarke 1960; Cotton 1974). Leaves and stems of taxa, covered with a layer of dense hairs, were removed using a scalpel.

Moreover, the taxa covered with many waxes, such as *I. oblongifolia* Forssk., *I. sessiliflora* DC*.*, and *I. colutea* (Burm.f.) Merr were treated with xylene in a test tube to melt the wax, then subjected to a warm water bath for 2–10 min at 60°C, and then transferred to lactic acid. The prepared slides were examined under 720 × and 1800 × magnifications with a Labomed light microscope powered by an ocular micrometre and a stage (Labo. America, Inc., USA). Line drawings were made using a Camera Lucida (PZO Microscope 10 × Drawing Eyepiece, Poland) with appropriate drawing scale bars to support the description of epidermal cells and stomata.

### Scanning Electron Microscopy (SEM)

Patterns of the leaf cuticle surface and the stem surface were studied by scanning electron microscopy (SEM). Samples of the mid-lamina position of dry leaves and the second upper internode of the stem were mounted onto clean stubs using double-sided adhesive tape. Samples were coated with gold using an SPI-Module ion-sputtering device and then examined and photographed using a JOEL JSM-5500 LV Scanning Electron Microscope (at an accelerating voltage of 10–15 kV) at the Central Laboratory of South Valley University, Qena, Egypt.

### Imaging and statistical analysis

Quantitative measurements were performed digitally using ImageJ v1.45 (Schneider et al. 2012). Stomata and epidermal cell frequencies were expressed as their numbers per micro-field area of the leaf from photos taken at 720 × magnification. Micrographs of both adaxial and abaxial surfaces were taken using a Leica DM500 light microscope with a digital video camera Leica ICC 50 (Leica Microsystems, Schweiz. AG., Switzerland). The counts were obtained from 30 readings to calculate the average values and estimate the stomatal index (SI) percentage by the formula described by Salisbury (1927):

$$SI=\frac{s}{s+E}\times 100$$

where: ***S*** denotes the number of stomata per unit area, and ***E*** is the number of epidermal cells in the same unit area. The values for the adaxial and abaxial epidermal surfaces were determined separately. We considered stoma types predominant when they covered ≥ 70% of the observed epidermal surface.

### Identification of stomatal complexes

We described the types of stomata, their distribution, and orientation according to the available literature (Metcalfe and Chalk 1950; Siu and Reese 1953; Inamdar and Chohan 1969a; Baranova 1972, 1992; Fryns-Claessens and Van Cotthem 1973; Dilcher 1974; Prabhakar 2004; Carpenter 2005; Mandal et al. 2012; Ghahremaninejad et al. 2012; Badry 2018; Ullah et al. 2018; Grohar et al. 2022).

## Results

**Fig. 1:**
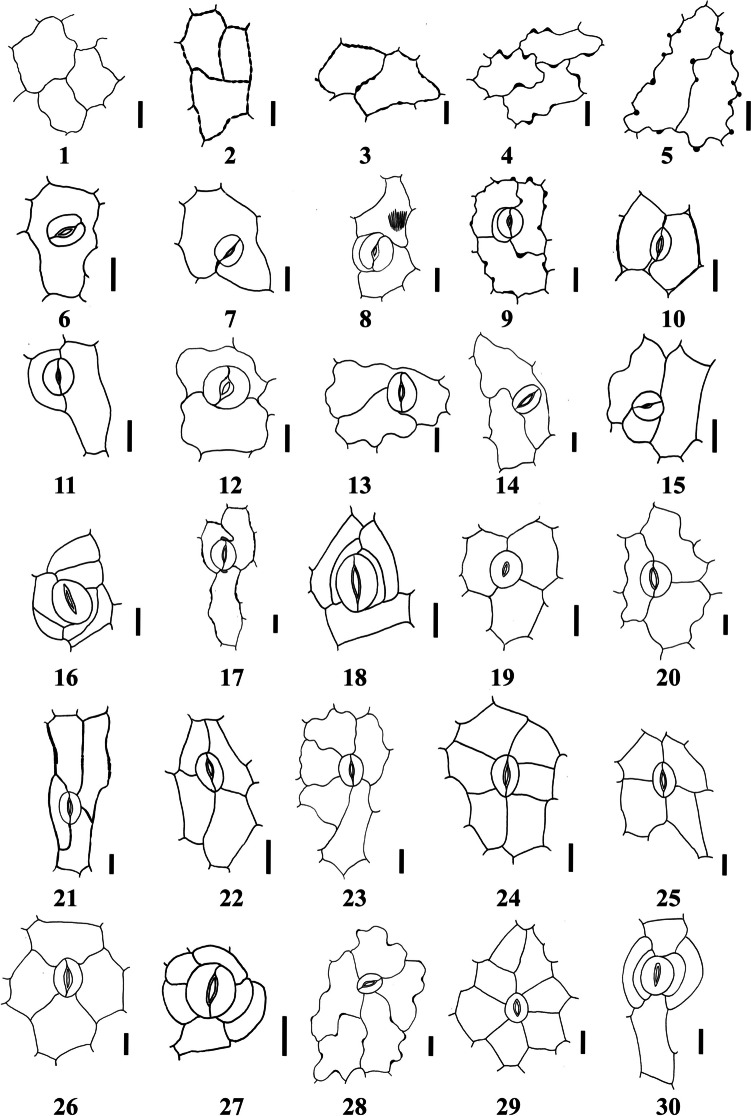
Camera Lucida illustrations of normal stomatal types and anticlinal wall thickness in the studied *Indigofera* taxa. (1–5) uniformity of thickness of the anticlinal wall; 1: smooth (*I. spiniflora* [leaf abaxial]). 2: beaded (*I. sessiliflora* [leaf adaxial]). 3: irregularly thickened (*I. colutea* [leaf abaxial]). 4: ridged (*I. subulata* var. *subulata* [leaf abaxial]). 5: knobbed (*I. subulata* var. *subulata* [leaf abaxial]). (6–30) normal stomatal types; 6: pericytic (*I. hochstetteri* [leaf abaxial]). 7: desmocytic (*I. spinosa* [leaf adaxial]). 8: hemiparacytic subtype-I (*I. arabica* [leaf abaxial]). 9: hemiparacytic subtype-II (*I. subulata* var. *subulata* [leaf abaxial]). 10: paracytic subtype-I (*I. cordifolia* [leaf abaxial]). 11: paracytic subtype-II (*I. hochstetteri* [leaf abaxial]). 12: diacytic (*I. arabica* [leaf adaxial]). 13: intermediate type between paracytic and diacytic (*I. spinosa* [leaf adaxial]). 14: polocytic (*I. spinosa* [leaf adaxial]). 15: anisocytic (*I. spiniflora* [leaf abaxial]). 16: amphianisocytic (*I. trita* [stem]). 17: anisotricytic (*I. colutea* [leaf adaxial]). 18: amphianisotricytic (*I. hochstetteri* [stem]). 19: isotricytic (*I. coerulea* [leaf abaxial]). 20: laterocytic subtype-I (*I. arabica* [leaf adaxial]). 21: laterocytic subtype-II (*I. subulata* var. *subulata* [leaf abaxial]). 22: laterocytic subtype-III (*I. oblongifolia* [leaf adaxial]). 23: laterocytic subtype- IV (*I. spiniflora* [leaf adaxial]). 24: laterocytic subtype-V (*I. oblongifolia* [leaf adaxial]). 25: staurocytic (*I. hochstetteri* [leaf abaxial]). 26: tetracytic (*I. trita* [leaf adaxial]). 27: cyclocytic (*I. colutea* [stem]). 28: anomocytic (*I*. *spinosa* [leaf adaxial]). 29: stephanocytic (*I. articulata [*leaf abaxial]). 30: hexacytic (*I. hochstetteri* [stem]). Scale bars = 50μm

**Fig. 2:**
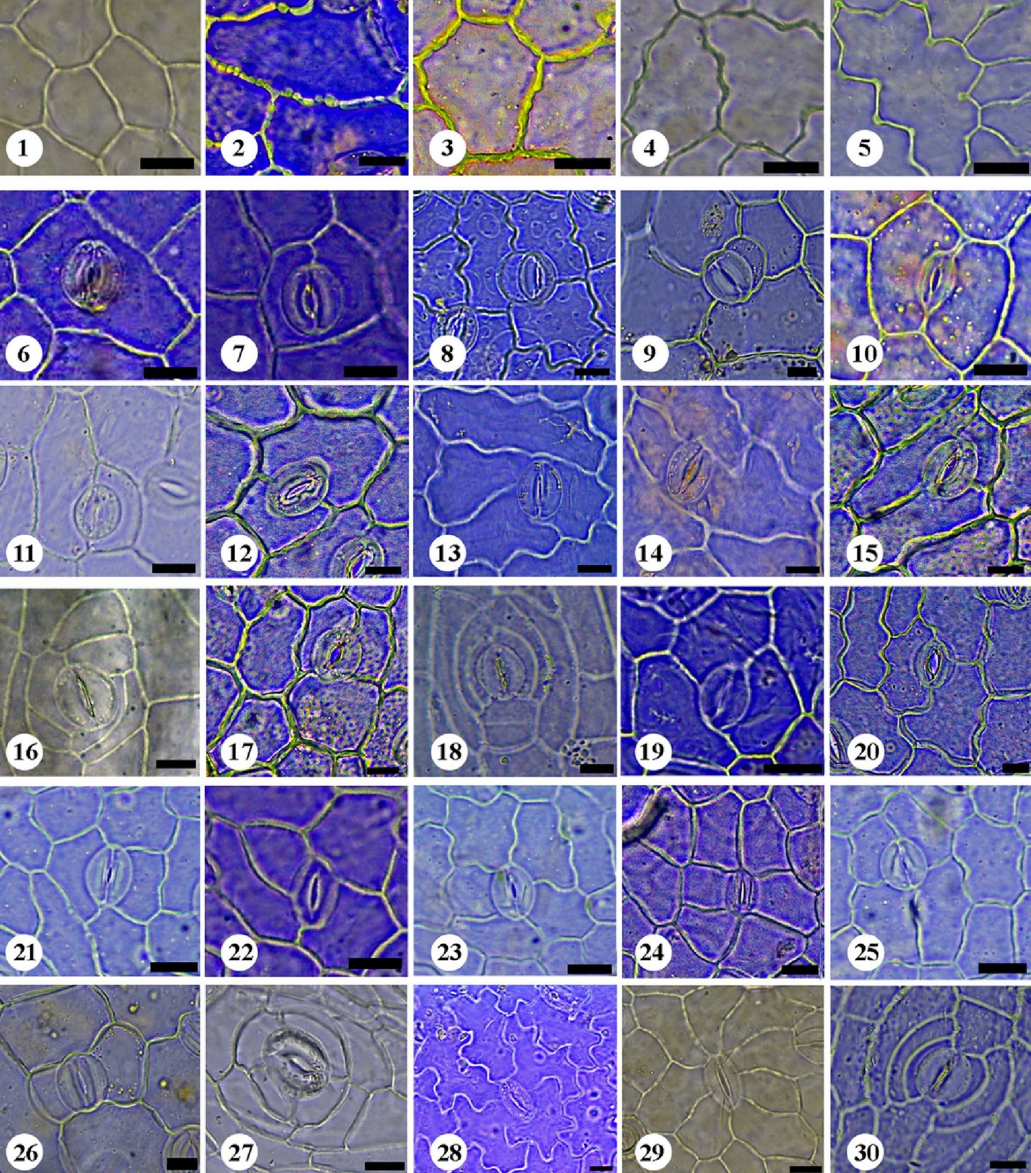
LM micrographs of epidermal peels of the studied taxa of *Indigofera.* (1–5) showing uniformity of thickness of the anticlinal wall; 1: **smooth** (*I. articulata* [leaf abaxial]). 2: **beaded** (*I. sessiliflora* [leaf abaxial]). 3: **irregularly thickened** (*I. colutea* [leaf abaxial]**)**. 4: **ridges** (*I. subulata* var. *subulata* [leaf abaxial])**.** 5**: knobs** (*I. spinosa* [leaf abaxial]). (6–30) showing normal stomatal types; 6: **pericytic** (*I. hochstetteri* [leaf abaxial]). 7: **desmocytic** (*I. hochstetteri* [leaf abaxial]). 8: **hemiparacytic subtype-I** (*I. hochstetteri* [leaf adaxial]). 9: **hemiparacytic subtype-II** (*I. trita* [leaf abaxial]). 10: **paracytic subtype-I** (*I. cordifolia* [leaf abaxial]). 11: **paracytic subtype-II** (*I. hochstetteri* [leaf abaxial]). 12: **diacytic** (*I. arabica* [leaf adaxial]). 13: **intermediate type between paracytic and diacytic** (*I. spinosa* [leaf adaxial]). 14: **polocytic** (*I. spinosa* [leaf adaxial]). 15: **anisocytic** (*I. arabica* [leaf adaxial]). 16: **amphianisocytic** (*I. trita* [stem]). 17: **anisotricytic** (*I. arabica* [leaf adaxial]). 18: **amphianisotricytic** (*I. hochstetteri* [stem]). 19: **isotricytic** (*I. oblongifolia* [leaf adaxial]). 20: **laterocytic subtype-I** (*I. arabica* [leaf adaxial]). 21: **laterocytic subtype-II** (*I. spiniflora* [leaf adaxial]). 22: **laterocytic subtype-III** (*I. oblongifolia* [leaf adaxial]). 23: **laterocytic subtype-IV** (*I. spiniflora* [leaf adaxial]). 24: **laterocytic subtype-V** (*I. oblongifolia* [leaf adaxial]). 25: **staurocytic** (*I. hochstetteri* [leaf abaxial]). 26: **tetracytic** (*I. trita* [leaf abaxial]). 27: **cyclocytic** (*I. arabica* [stem]). 28: **anomocytic** (*I*. *spinosa* [leaf abaxial]). 29: **stephanocytic** (*I. articulata ***[**leaf abaxial]). 30: **hexacytic** (*I. hochstetteri* [stem]). Scale bars = 10μm

**Fig. 3:**
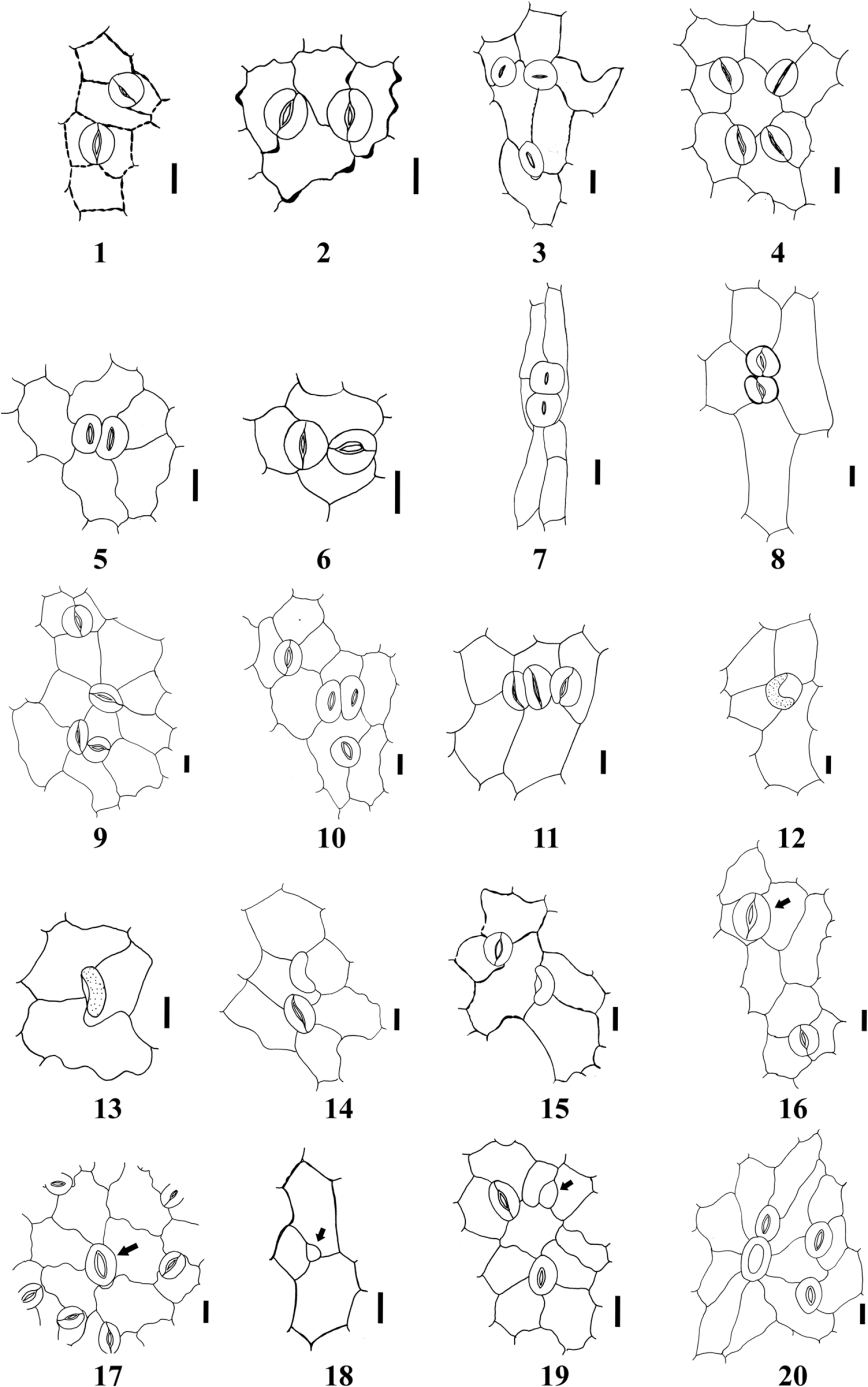
Camera Lucida illustrations of abnormal stomatal types in the studied taxa of *Indigofera*. 1: contiguous stomata subtype-I (*I. sessiliflora* [leaf adaxial]). 2: contiguous stomata subtype-II (*I. subulata* var. *subulata* [leaf adaxial]). 3: contiguous stomata subtype-III (*I. sessiliflora* [leaf abaxial]). 4: contiguous stomata subtype-IV (*I. hochstetteri* [leaf adaxial]). 5: twin stomata subtype-I (*I. articulata* [leaf adaxial]). 6: twin stomata subtype-II (*I. hochstetteri* [leaf adaxial]). 7–8: twin stomata subtype-III (7: *I. articulata* [stem]; 8: *I. trita* [leaf abaxial]). 9–10: twin stomata subtype-IV (9: *I. trita* [leaf abaxial]; 10: *I. sessiliflora* [leaf abaxial]). 11: triple stomata (*I. trita* [leaf abaxial]). 12: single guard cell subtype-I (*I. trita* [leaf abaxial]).13: single guard cell subtype-II (*I. spinosa* [leaf adaxial]).14–15: single guard cell subtype-III (14: *I. trita* [leaf adaxial]; 15: *I. sessiliflora* [leaf abaxial]). 16: Giant stoma (arrow) (*I. arabica* [leaf adaxial]). 17: Giant stoma subtype-I (arrow) (*I. hochstetteri* [leaf abaxial]). 18: arrested stomata (arrow) (*I. cordifolia* [leaf abaxial]). 19: arrested stomata subtype-I (arrow) (I. *articulata* [leaf abaxial]). 20: stoma associated with foot cell (*I. arabica* [leaf abaxial]). Scale bars = 50μm

**Fig. 4:**
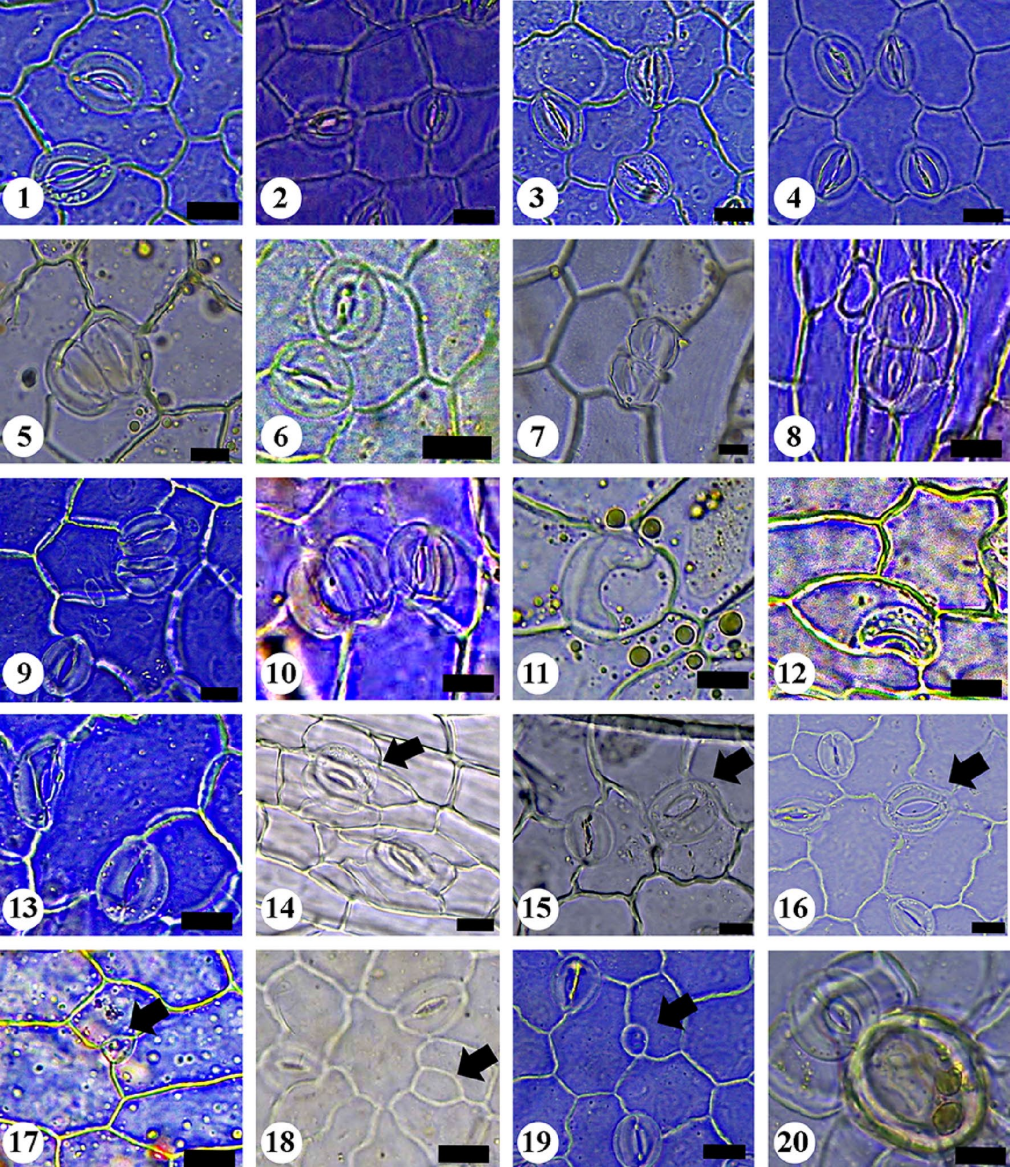
LM micrographs of epidermal peels of the studied taxa of *Indigofera* showing abnormal stomatal types. 1: contiguous stomata subtype -I (*I. hochstetteri* [leaf adaxial]). 2: contiguous stomata subtype -II (*I. hochstetteri* [leaf abaxial]). 3: contiguous stomata subtype -III (*I. hochstetteri* [leaf adaxial]). 4: contiguous stomata subtype -IV (*I. hochstetteri* [leaf adaxial]). 5: twin stomata subtype -I (*I. trita* [leaf abaxial). 6: twin stomata subtype -II (*I. hochstetteri* [leaf adaxial]). 7–8: twin stomata subtype -III (7: *I. trita* [leaf abaxial; 8: *I. articulata* [stem]]). 9: twin stomata subtype -IV (9: *I. sessiliflora* [leaf abaxial]). 10: triple stomata (*I. trita* [leaf abaxial]). 11: single guard cell subtype-I (*I. trita* [leaf abaxial]).12: single guard cell subtype-II (*I. arabica* [leaf abaxial]).13: single guard cell subtype-III (*I. sessiliflora* [leaf abaxial]). 14: Giant stoma (arrow) (I. *articulata* [stem]). 15–16: Giant stoma subtype-I (arrow) (15: *I. arabica* [leaf adaxial; 16: *I. hochstetteri* [leaf abaxial]). 17: arrested stomata (arrow) (*I. cordifolia* [leaf abaxial]). 18–19: arrested stomata subtype-I (arrow) (18: I. *articulata* [leaf abaxial]; 19: *I. hochstetteri* [leaf adaxial]). 20: stoma associated with foot cell (*I. trita* [leaf abaxial]). Scale bars = 10μm

**Fig. 5:**
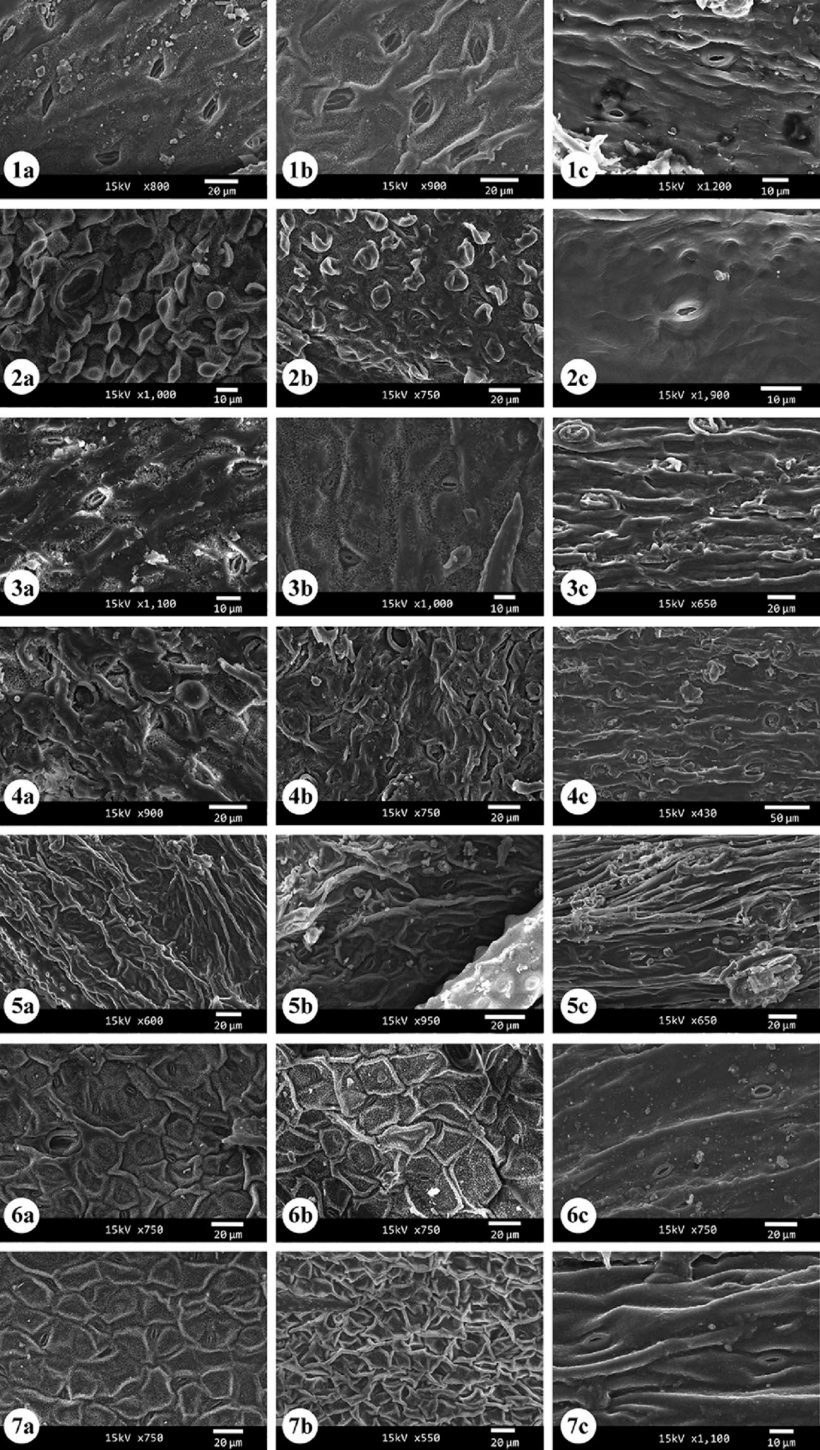
SEM micrographs of stomata and leaf surface patterns in the studied taxa of *Indigofera*. **a** Adaxial surface; **b** Abaxial surface; **c** stem surface. (1) *I. arabica*; (2) *I. argentea* (papillae on abaxial and adaxial surfaces*);* (3) *I. articulata*; (4) *I. coerulea*; (5) *I. colutea*; (6) *I. cordifolia*, (7) *I. hochstetteri*

**Fig. 6:**
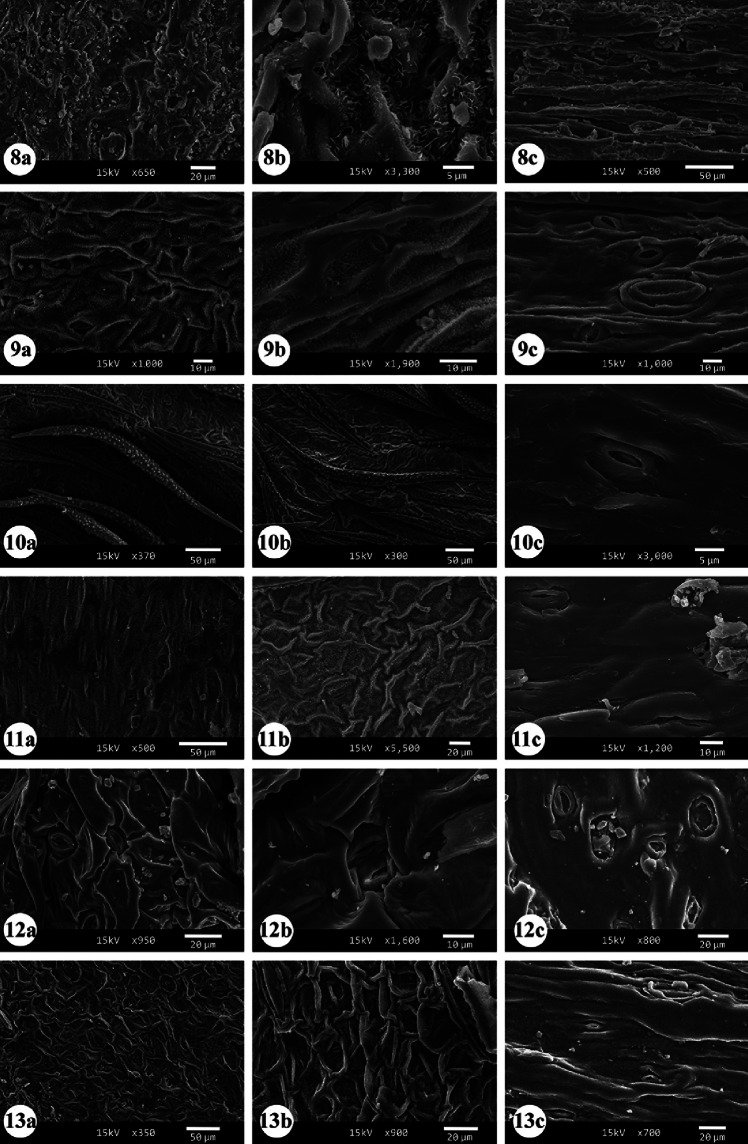
SEM micrographs of stomata and leaf surface patterns in the studied taxa of *Indigofera*. **a** Adaxial surface; **b** Abaxial surface; **c** stem surface. (8) *I. oblongifolia*; (9) *I. sessiliflora*; (10) *I. spiniflora;* (11) *I. spinosa*; (12) *I. trita*; (13) *I. subulata* var. *subulata*

The quantitative and qualitative characteristics of epidermal cells and stomata were studied on the adaxial and abaxial leaf surfaces and the stem of the examined taxa (Tables 2, 3, 4 and 5). Selected micrographs showing the micro-morphological characteristics are presented in Figs. 1, 2, 3, 4, 5 and 6.

**Table 2:** Qualitative micro–morphological characteristics of epidermal cells and stomata in leaves and stems of the studied taxa of *Indigofera*

Taxa/ Character	Leaf / stem surface	Epidermal cell shape	Epidermal cell wall	Uniformity of thickness of anticlinal wall	Leaf cuticle surface	Crystal type	Stomatal shape	Stomatal position	Stomatal ends in outline
*Indigofera arabica* Jaub. & Spach	AD	Irregular, tetragonal, pentagonal, hexagonal, polygonal	Slightly undulate	Smooth	Rugulate	Raphides	Circular, elliptic to widely elliptic, oblong	Deeply sunken	Rounded to retuse
	AB	Irregular, tetragonal, pentagonal, hexagonal, polygonal	Undulate	Smooth	Rugulate	Raphides	Circular, elliptic to widely elliptic, oblong	Sunken	Rounded to retuse
	ST	Tetragonal, pentagonal, hexagonal polygonal, elongate	Slightly undulate to arched	Smooth	NA	**-**	Circular, widely elliptic, oblong	At the same level	Rounded to retuse
*Indigofera argentea* Burm.f	AD	Irregular, tetragonal, pentagonal, hexagonal, polygonal	Straight to slightly undulate	Smooth to ridges	Ruminate	**-**	Elliptic to widely elliptic, oblong	Deeply sunken	Rounded to retuse
	AB	Irregular, tetragonal, pentagonal, hexagonal, polygonal	Stright to slightly undulate	Smooth to ridges	Reticulate	**-**	Elliptic to widely elliptic, oblong	Sunken to deeply sunken	Rounded to retuse
	ST	Irregular, tetragonal, pentagonal, polygonal	Straight to arched	Smooth to beaded	NA	**-**	Widely elliptic, oblong	Slightly sunken	Rounded to retuse
*Indigofera articulata* Gouan	AD	Irregular, tetragonal, pentagonal, hexagonal, polygonal	Straight, slightly undulate to undulate	Smooth	Rugulate	**-**	Elliptic to widely elliptic, oblong	Slightly sunken	Rounded to retuse
	AB	Irregular, tetragonal, pentagonal, hexagonal, polygonal	Stright, slightly undulate	Smooth	Rugulate	**-**	Elliptic to widely elliptic, oblong	Slightly sunken	Rounded to retuse
	ST	Tetragonal, pentagonal, hexagonal, elongate	Straight to slightly undulate	Smooth	NA	**-**	Circular, widely elliptic, rectangular	At the same position to slightly raised	Rounded to retuse
*Indigofera coerulea* var. *coerulea* Roxb	AD	Tetragonal, pentagonal, hexagonal, and polygonal	Straight to slightly undulate	Smooth	Rugulate	**-**	Circular, elliptic to widely elliptic	Slightly sunken to sunken	Rounded
	AB	Tetragonal, pentagonal, hexagonal, Polygonal	Straight to slightly arched	Smooth	Rugulate	**-**	Circular, elliptic to widely elliptic	At the same level, slightly sunken	Rounded
	ST	Tetragonal, pentagonal, hexagonal, polygonal, elongate	Straight	Smooth	NA	**-**	Circular, widely elliptic	At the same level	Rounded
*Indigofera colutea* (Burm.f.) Merr	AD	Irregular, tetragonal, pentagonal hexagonal	Straight t to slightly undulate	Smooth to beaded, irregularly thickened	Rugulate	**-**	Elliptic to widely elliptic, oblong	Slightly sunken to sunken	Rounded to retuse
	AB	Irregular, tetragonal, pentagonal hexagonal, polygonal	Straight to slightly undulate	Smooth to beaded, irregularly thickened	Rugulatetoruminate	**-**	Elliptic to widely elliptic, circular	Slightly sunken to sunken	Rounded to retuse
	ST	Irregular, tetragonal, pentagonal, hexagonal, elongate	Straight to arched	Smooth	NA	**-**	Elliptic to widely elliptic, oblong, circular	At the same level	Rounded to retuse
*Indigofera cordifolia* B. Heyne ex Roth	AD	Irregular, tetragonal, pentagonal, hexagonal, polygonal	Straight to slightly undulate	Smooth to beaded, irregularly thickened	Reticulate	**-**	Elliptic to narrow elliptic, oblong	Slightly sunken to sunken	Rounded
	AB	Irregular, tetragonal, pentagonal, hexagonal, polygonal	Straight to slightly undulate	Smooth to beaded	Reticulate	**-**	Elliptic to narrow elliptic	Slightly sunken to sunken	Rounded
	ST	Tetragonal, pentagonal, hexagonal, elongate	Straight to rounded	Smooth to beaded	NA	**-**	Elliptic to widely elliptic	At the same level	Rounded
*Indigofera hochstetteri* Baker	AD	Irregular, tetragonal, pentagonal, hexagonal, polygonal	Straight t to slightly undulate	Smooth to ridges	Ruminate	**-**	Elliptic to widely elliptic, oblong	Slightly sunken	Rounded to retuse
	AB	Irregular, tetragonal, pentagonal, hexagonal, polygonal	Straight to slightly undulate	Smooth to beaded	Ruminate	**-**	Elliptic to widely elliptic, oblong, circular	Sunken	Rounded to retuse
	ST	Tetragonal, pentagonal, hexagonal, elongate	Straight to arched	Smooth to beaded	NA	**-**	Elliptic to widely elliptic, oblong, circular	At the same level	Rounded
*Indigofera oblongifolia* Forssk	AD	Tetragonal, pentagonal, hexagonal, polygonal	Straight to slightly arched	Smooth	Rugulate to ruminate	**-**	Elliptic to widely elliptic, oblong	Deeply sunken	Rounded to retuse
	AB	Tetragonal, pentagonal, hexagonal, polygonal	Straight to slightly arched	Smooth	Rugulate	**-**	Elliptic to widely elliptic	Slightly sunken	Rounded to retuse
	ST	Tetragonal, pentagonal, hexagonal	Straight	Smooth to irregularly thickened	NA	**-**	Widely elliptic, oblong	Sunken	Rounded
*Indigofera sessiliflora* DC	AD	Irregular, tetragonal, pentagonal, hexagonal, polygonal	Straight to slightly undulate	Smooth to beaded, irregularly thickened	Ruminate	**-**	Elliptic to widely elliptic, circular, oblong	Slightly to deeply sunken	Rounded to retuse
	AB	Irregular, tetragonal, pentagonal, hexagonal, polygonal	Straight to slightly undulate	Smooth to beaded,irregularly thickened	Rugulate	**-**	Elliptic to widely elliptic, oblong	Slightly sunken to sunken	Rounded to retuse
	ST	Tetragonal, pentagonal, hexagonal, polygonal, elongate	Straight to slightly arched	Smooth to beaded	NA	**-**	Elliptic to widely elliptic, circular, oblong	At the same level to slightly sunken	Rounded
*Indigofera spiniflora* Hochst. ex Boiss	AD	Irregular, tetragonal, pentagonal, hexagonal, polygonal	Straight, slightly undulate to undulate	Smooth to beaded	Ruminate	**-**	Elliptic to widely elliptic, oblong	Sunken	Rounded to retuse
	AB	Irregular, tetragonal, pentagonal, hexagonal, polygonal	Straight, slightly undulate)	Smooth to beaded,irregularly thickened	Rugulate	**-**	Elliptic to widely elliptic, oblong	At the same level	Rounded to retuse
	ST	Tetragonal, pentagonal, hexagon, polygonal, elongate	Straight to arched	Smooth to beaded	NA	**-**	Elliptic to widely elliptic, circular	At the same level	Rounded to retuse
*Indigofera spinosa* Forssk	AD	Irregular, tetragonal, pentagonal, polygonal	Undulate to deeply undulate	Smooth,ridges,beaded	Rugulate toruminate	**-**	Elliptic to widely elliptic, oblong	Slightly sunken to sunken	Rounded to retuse
	AB	Irregular, tetragonal	Undulate to deeply undulate	Smooth to ridges to beaded	Ruminate	-	Elliptic, oblong	Slightly sunken to sunken	Rounded to retuse
	ST	Tetragonal, hexagonal, elongate	Straight to arched	Smooth	NA	**-**	Widely elliptic, circular, oblong	Slightly sunken	Rounded to retuse
*Indigofera subulata* Vahl ex Poir. var.* subulata*	AD	Irregular, tetragonal, pentagonal, hexagonal, polygonal	Undulate to deeply undulate	Ridges to knobs	Reticulate	**-**	Elliptic to widely elliptic, circular, oblong	Slightly sunken	Rounded to retuse
	AB	Irregular, tetragonal, pentagonal, hexagonal, polygonal	Undulate to deeply undulate	Ridges to knobs	Reticulate	**-**	Elliptic to widely elliptic, oblong, circular	Slightly sunken	Rounded to retuse
	ST	Tetragonal, pentagonal, hexagonal polygonal	Straight to arched	Smooth to beaded	NA	**-**	Elliptic to widely elliptic, oblong	At the same level	Rounded to retuse
*Indigofera trita* L. F	AD	Irregular, tetragonal, pentagonal, hexagonal, polygonal	Straight -to slightly undulate	Smooth	Ruminate	**-**	Elliptic to widely elliptic, oblong	At the same level to slightly sunken	Rounded to retuse
	AB	Irregular, tetragonal, pentagonal, hexagonal, polygonal	Straight to slightly undulate	Smooth	Rugulate	Solitary	Elliptic to widely elliptic, circular, oblong	At the same level to slightly sunken	Rounded to retuse
	ST	Tetragonal, pentagonal, hexagonal, polygonal, elongate	Straight to slightly arched	Smooth to beaded	NA	**-**	Elliptic to widely elliptic, circular, oblong	At the same level	Rounded to retuse

Abbreviations: AD: adaxial leaf surface, AB: abaxial leaf surface, ST: stem surface, NA: non-applicable

On the other hand, the epidermal cells of the stems were primarily tetragonal, pentagonal, hexagonal, polygonal, and elongated with straight to arched or slightly undulate anticlinal wall outlines. Polygonal cell shape of the epidermal cells was present in all species except *I. articulata* Gouan, *I. oblongifolia*, *I. hochstetteri* Baker, and *I. spinosa*, and irregular form is present in *I. argentea* Burm.f. and *I. colutea* (Figs. 1, 2, 3, 4, 5, 6 and Table 2). The foliar epidermal cell frequency showed a great diversity among the taxa. The epidermal cell frequency was highest on the upper surface of *I. oblongifolia* with an average frequency of 196.80 and lowest on the lower surface of *I. trita* with an average frequency of 49.17. Table 3 shows the detailed counts for the epidermal cell frequencies among the studied taxa.

**Table 3:** Quantitative micro-morphological characteristics of stomata and epidermal cells in the leaves of the studied taxa of *Indigofera*

Taxa/Character	Leaf surface	Stomata dimensions L × W (µm) mean (min–max)	Stomatal pore length (µm) mean (min–max)	Stomatal pore width (µm) mean (min–max)	Epidermal cells frequency counts/field	Stomatal cells frequency counts/field	Stomatal index (%) mean (min–max)
*Indigofera arabica* Jaub. & Spach	AD	25.23 (20.90–34.80) × 21.26 (15.49–24.75)	11.03 (6.05–14.62)	3.22 (1.25–5.66)	67.90 (59.00–82.00)	15.98 (12.00–21.00)	19.05 (15.29–23.38)
	AB	23.64 (16.61–29.64) × 20.12 (16.42–26.98)	10.72 (4.19–21.84)	2.09 (0.42–5.83)	72.63 (60.00–121.00)	16.77 (14.00–24.00)	18.87 (12.32–23.08)
*Indigofera argentea* Burm.f	AD	18.16 (15.36–22.04) × 15.88 (13.01–19.68)	7.12 (2.11–10.99)	1.63 (0.85–2.78)	127.20 (106.00–150.00)	19.83 (12.00–26.00)	13.42 (9.30–16.54)
	AB	18.14 (15.05–22.60) × 15.90 (13.49–18.05)	7.29 (1.87–12.12)	1.68 (0.60–3.94)	122.10 (88.00–159.00)	24.97 (11.00–29.00)	16.22 (9.45–50.21)
*Indigofera articulata* Gouan	AD	19.65 (16.24–22.52) × 16.15 (14.48–18.01)	7.77 (3.52–10.65)	1.14 (0.57–2.11)	124.53 (109.00–154.00)	17.57 (11.00–25.00)	12.40 (8.09–16.78)
	AB	20.49 (16.68–28.01) × 15.58 (12.37–17.90)	9.09 (4.36–16.25)	1.66 (0.68–3.25)	159.27 (138.00–185.00)	20.87 (15.00–28.00)	13.14 (10.07–17.39)
*Indigofera coerulea* var. *coerulea* Roxb	AD	19.24 (15.80–25.05) × 16.64 (14.87–19.49)	7.25 (5.09–10.36)	1.48 (0.56–2.34)	156.47 (139.00–184.00)	27.97 (19.00–34.00)	15.15 (11.11–18.38)
	AB	18.48 (14.62–22.16) × 15.52 (13.89–17.51)	6.60 (2.31–9.87)	1.41 (0.34–2.84(	155.20 (145.00–174.00	22.37 (19.00–29.00)	12.59 (10.77–14.65)
*Indigofera colutea* (Burm.f.) Merr	AD	21.20 (16.05–25.17) × 19.21 (11.98–25.31)	8.25 (4.67–16.31)	1.46 (0.58–3.29)	97.40 (88.00–117.00)	20.53 (16.00–27.00(	17.39 (14.29–21.93)
	AB	21.17 (18.00–25.40) × 19.31 (17.40–22.18)	9.53 (5.69–13.28)	1.75 (0.57–5.22	83.23 (68.00–104.00)	16.60 (11.00–25.00)	16.57 (12.09–22.33)
*Indigofera cordifolia* B. Heyne ex Roth	AD	13.80 (12.14–15.41) × 10.35 (8.20–13.65)	5.26 (3.53–10.84)	1.02 (0.60–1.76)	95.83 (76.00–128.00)	21.07 (14.00–28.00)	18.06 (11.11–22.94)
	AB	15.38 (13.57–17.92) × 10.84 (7.93–12.12)	5.89 (3.44–8.26)	1.02 (0.36–1.61)	86.90 (70.00–108.00)	19.70 (10.00–25.00)	18.52 (9.62–23.08)
*Indigofera hochstetteri* Baker	AD	18.43 (13.95–21.77) × 16.11 (13.17–18.21)	6.86 (3.84–10.59)	1.34 (0.57–2.26)	102.07 (90.00–120.00)	38.67 (29.00–48.00)	27.46 (22.14–32.65)
	AB	20.41 (16.54–26.35) × 15.99 (13.30–18.49)	8.66 (5.17–12.91)	1.37 (0.67–2.72)	107.37 (76.00–137.00)	18.43 (13.95–21.77)	26.01 (20.95–30.28)
*Indigofera oblongifolia* Forssk	AD	16.71 (12.87–20.65) × 12.95 (10.18–15.92)	7.15 (4.45–10.12)	1.09 (0.49–2.12)	174.63 (138.00–201.00)	29.20 (19.00–36.00)	14.36 (10.00–17.86)
	AB	18.11 (15.11–21.62) × 13.10 (11.08–15.34)	7.79 (5.18–11.59)	1.36 (0.75–3.18)	196.80 (106.00–243.00	21.47 (13.00–28.00)	9.95 (5.99–14.52)
*Indigofera sessiliflora* DC	AD	20.06 (15.75–25.24) × 15.67 (13.73–17.67)	8.87 (4.69–15.20)	1.61 (0.67–2.97)	90.57 (68.00–112.00)	31.80 (22.00–39.00)	25.98 (19.82–29.69)
	AB	26.03 (19.01–33.85) × 20.10 (15.21–25.46)	14.09 (6.63–23.64)	2.20 (0.88–4.52)	65.37 (45.00–80.00)	24.37 (18.00–29.00)	27.19 (23.26–31.88)
*Indigofera spiniflora* Hochst. ex Boiss	AD	18.40 (14.93–21.94) × 15.74 (13.69–18.39)	7.17 (4.72–10.37)	2.19 (0.91–3.66)	144.50 (95.00–212.00)	34.53 (16.00–48.00)	18.01 (11.54–23.13)
	AB	19.05 (14.81–23.09) × 15.62 (12.35–17.89)	8.19 (3.39–12.2 5)	1.85 (0.62–2.95)	128.47 (109.00–159.00)	29.23 (21.00–40.00)	18.46 (15.56–22.62)
*Indigofera spinosa* Forssk	AD	25.39 (18.23–31.49) × 17.97 (15.45–21.38)	11.93 (7.51–17.15)	1.36 (0.38–2.98)	54.03 (43.00–64.00)	9.37 (6.00–12.00)	14.81 (9.38–20.37)
	AB	27.63 (22.01–34.56) × 17.12 (15.26–24.85)	15.91 (10.2326.19)	2.25 (0.96–4.61)	62.10 (43.00–75.00)	9.63 (6.00–14.00)	13.39 (10.39–18.52)
*Indigofera subulata* Vahl ex Poir. var. *subulata*	AD	20.18 (16.99–23.41) × 17.30 (14.92–18.96)	7.98 (4.86–11.19)	1.69 (0.69–3.85)	91.13 (76.00–102.00)	21.87 (14.00–26.00)	19.30 (15.38–22.61)
	AB	20.57 (15.83–26.37) × 17.32 (14.38–20.21)	9.03 (4.58–14.29)	2.28 (0.74–4.68)	86.30 (67.00–109.00)	18.10 (11.00–66.00)	16.73 (11.34–42.86)
*Indigofera trita* L. F	AD	25.36 (19.86–33.92) × 20.91 (14.82–26.32)	10.83 (6.57–17.16)	1.71 (0.77–3.20)	56.03 (50.00–64.00)	19.67 (15.00–25.00)	25.96 (22.73–32.89)
	AB	26.96 (19.39–34.01) × 23.72 (19.01–26.77)	10.21 (3.63–16.59)	1.74 (0.86–2.75)	49.17 (34.00–69.00)	14.10 (6.00–23.00)	22.08 (13.95–27.66)

Abbreviations: AD: adaxial leaf surface, AB: abaxial leaf surface

**Table 4:** Organographic distribution of various normal stomatal types in the studied taxa of *Indigofera*

Taxa/ Character	AD/AB/ST	Normal stomata																								
		Peri	Desmo	Hemipara		Para		Dia	ITBPD	Polo	Aniso	Amphianiso	Anisotri	Amphianisotri	Isotri	Latero					Stauro	Tetra	Cyclo	Anomo	Stephano	hexa
				S- I	S-II	S-I	S-II									S-I	S-II	S-III	S-IV	S -V						
*Indigofera arabica* Jaub. & Spach	AD	**-**	**-**	** + **	** + **	**-**	** + **	** + **	** + **	**-**	** + **	**-**	** + **	**-**	** + **	** + **	** + **	**-**	** + **	**-**	** + **	** + **	**-**	** + **	** + **	**-**
	AB	**-**	**-**	** + **	** + **	** + **	**-**	**-**	** + **	**-**	** + **	**-**	** + **	**-**	** + **	** + **	** + **	** + **	**-**	**-**	** + **	** + **	**-**	** + **	** + **	**-**
	ST	**-**	**-**	**-**	**-**	**-**	** + **	**-**	**-**	**-**	** + **	** + **	** + **	** + **	** + **	**-**	** + **	**-**	**-**	**-**	**-**	** + **	** + **	** + **	**-**	**-**
*Indigofera argentea* Burm.f	AD	**-**	**-**	**-**	**-**	**-**	**-**	**-**	**-**	**-**	** + **	**-**	** + **	**-**	** + **	** + **	**-**	**-**	**-**	**-**	** + **	** + **	**-**	** + **	** + **	**-**
	AB	**-**	**-**	**-**	**-**	**-**	**-**	**-**	**-**	**-**	** + **	**-**	** + **	**-**	** + **	**-**	**-**	** + **	** + **	**-**	**-**	** + **	**-**	** + **	** + **	**-**
	ST	**-**	**-**	**-**	** + **	**-**	**-**	**-**	**-**	**-**	** + **	**-**	** + **	**-**	** + **	**-**	**-**	** + **	** + **	**-**	** + **	** + **	** + **		**-**	**-**
*Indigofera articulata* Gouan	AD	**-**	**-**	**-**	**-**	** + **	** + **	**-**	** + **	**-**	** + **	**-**	** + **	**-**	** + **	** + **	** + **	**-**	**-**	**-**	** + **	** + **	**-**	** + **	** + **	**-**
	AB	**-**	**-**	** + **	** + **	** + **	** + **	**-**	**-**	**-**	** + **	**-**	** + **	**-**	**-**	** + **	**-**	**-**	** + **	**-**	** + **	** + **	**-**	**-**	**-**	**-**
	ST	**-**	**-**	**-**	** + **	**-**	**-**	**-**	**-**	**-**	** + **	**-**	** + **	**-**	** + **	**-**	**-**	**-**	** + **	**-**	** + **	** + **	** + **	** + **	** + **	**-**
*Indigofera coerulea* var. *coerulea* Roxb	AD	**-**	**-**	** + **	**-**	** + **	** + **	**-**	**-**	**-**	** + **	**-**	** + **	**-**	** + **	** + **	** + **	**-**	**-**	**-**	** + **	** + **	**-**	** + **	** + **	**-**
	AB	**-**	**-**	** + **	** + **	** + **	** + **	**-**	**-**	**-**	** + **	**-**	** + **	**-**	** + **	**-**	**-**	**-**	**-**	**-**	** + **	** + **	**-**	** + **	** + **	**-**
	ST	**-**	**-**	**-**	** + **	**-**	**-**	**-**	** + **	**-**	** + **	**-**	** + **	**-**	**-**	**-**	**–**	**-**	**-**	**-**		** + **	**-**		**-**	**-**
*Indigofera colutea* (Burm.f.) Merr	AD	**-**	**-**	** + **	** + **	** + **	** + **	**-**	**-**	**-**	** + **	**-**	** + **	**-**	** + **	**-**	** + **	**-**	**-**	**-**	** + **	** + **	**-**	** + **	**–**	**-**
	AB	**-**	**-**	**-**	**-**		** + **	**-**	** + **	**-**	** + **	**-**	** + **	**-**	**-**	**-**	**-**	**-**	**-**	**-**	** + **	** + **	**-**	** + **	** + **	**-**
	ST	**-**	**-**	**-**	**-**	** + **	** + **	**-**	**-**	**-**	** + **	**-**	** + **	**-**	** + **	** + **	**-**	**-**	**-**	**-**	** + **	** + **	** + **	** + **	** + **	** + **
*Indigofera cordifolia* B. Heyne ex Roth	AD	**-**	**-**	** + **	** + **	** + **	** + **	**-**	** + **	**-**	** + **	**-**	** + **	**-**	** + **	** + **	**-**	**-**	**-**	**-**	** + **	** + **	**-**	** + **	**-**	**-**
	AB	**-**	**-**	** + **	**-**	** + **	** + **	**-**	** + **	**-**	** + **	**-**	** + **	**-**	** + **	**-**	**-**	**-**	**-**	**-**	** + **	** + **	**-**	**-**	**-**	**-**
	ST	**-**	**-**	** + **	**-**	** + **	** + **	**-**	** + **	**-**	** + **	**-**	** + **	**-**	** + **	** + **	**-**	**-**	**-**	**-**	** + **	** + **	** + **	** + **	**-**	**-**
*Indigofera hochstetteri* Baker	AD	**-**	**-**	** + **	** + **	**-**	** + **	**-**	**-**	** + **	** + **	**-**	**-**	**-**	** + **	**-**	**-**	**-**	** + **	**-**	**-**	** + **	**-**	** + **		**-**
	AB	** + **	** + **	**-**	**-**	**-**	** + **	**-**	**-**	**-**	** + **	**-**	** + **	**-**	** + **	**-**	**-**	**-**	**-**	**-**	** + **	** + **	**-**	** + **	** + **	**-**
	ST	**-**	**-**	**-**	**-**	** + **	**-**	**-**	**-**	**-**	** + **	** + **	** + **	** + **	** + **	** + **	**-**	**-**	**-**	**-**	** + **	**-**	** + **	** + **	**-**	** + **
*Indigofera oblongifolia* Forssk	AD	**-**	**-**	** + **	**-**	**-**	**-**	**-**	**-**	**-**	** + **	**-**	** + **	**-**	** + **	** + **	** + **	** + **	**-**	** + **	** + **	** + **	**-**	** + **	** + **	**-**
	AB	**-**	**-**	** + **	**-**	**-**	**-**	**-**	**-**	**-**	** + **	**-**	** + **	**-**	** + **	** + **	**-**	**-**	** + **	**-**	** + **	** + **	**-**	** + **	** + **	**-**
	ST	**-**	**-**	**-**	**-**	**-**	**-**	**-**		**-**	** + **	**-**	** + **	**-**	** + **	**-**	**-**	**-**	**-**	**-**	**-**	** + **	**-**	** + **	** + **	**-**
*Indigofera sessiliflora* DC	AD	**-**	** + **	**-**	**-**	**-**	**-**	**-**	** + **	**-**	** + **	**-**	** + **	**-**	** + **	**-**	**-**	**-**	**-**	**-**	**-**	** + **	**-**	**-**	** + **	**-**
	AB	**-**	** + **	**-**	**-**	**-**	** + **	**-**	** + **	**-**	** + **	**-**	** + **	**-**	** + **	** + **	**-**	**-**	**-**	**-**	**-**	**-**	**-**	**-**	**-**	**-**
	ST	**-**	**-**	** + **	**-**	**-**	** + **	**-**	** + **	**-**	** + **	** + **	** + **	** + **	** + **	**-**	**-**	**-**	**-**	**-**	**-**	** + **	** + **	** + **	**-**	** + **
*Indigofera spiniflora* Hochst. ex Boiss	AD	**-**	**-**	** + **	** + **	**-**	** + **	**-**	**-**	**-**	** + **	**-**	** + **	**-**	** + **	** + **	** + **	**-**	**-**	**-**	** + **	** + **	**-**	** + **	** + **	**-**
	AB	**-**	**-**	**-**	**-**	**-**	** + **	**-**	**-**	**-**	** + **	**-**	** + **	**-**	** + **	**-**	** + **	**-**	**-**	**-**	** + **	**-**	**-**	** + **	** + **	**-**
	ST	**-**	**-**	**-**	**-**	**-**	**-**	**-**	**-**	**-**	** + **	**-**	** + **	**-**	**-**	** + **		**-**	**-**	**-**	** + **	** + **	** + **	** + **	** + **	**-**
*Indigofera spinosa* Forssk	AD	** + **	** + **	** + **	**-**	** + **	**-**	**-**	** + **	** + **	** + **	**-**	** + **	**-**	** + **	**-**	** + **	**-**	**-**	**-**	** + **	** + **	**-**	**-**	**-**	**-**
	AB	**-**	**-**	**-**	**-**	**-**	**-**	**-**	**-**		** + **	**-**	**-**	**-**	** + **	** + **	** + **	**-**	** + **	**-**	** + **	**-**	**-**	** + **	**-**	**-**
	ST	**-**	** + **	**-**	**-**	** + **	** + **	**-**	**-**	** + **	** + **	**-**	** + **	**-**	**-**	** + **	**-**	**-**	**-**	**-**	** + **	** + **	** + **	** + **	**-**	**-**
*Indigofera subulata* Vahl ex Poir. var.* subulata*	AD	**-**	**-**	** + **	**-**	**-**	** + **	**-**	**-**		** + **	**-**	** + **	**-**	** + **	** + **	** + **	**-**	**-**	**-**	** + **	** + **	**-**	** + **	** + **	**-**
	AB	**-**	**-**	** + **	** + **	** + **	** + **	**-**	**-**	** + **	** + **	**-**	** + **	**-**	** + **	** + **	** + **	**-**	**-**	**-**	** + **	** + **	**-**	** + **	**-**	**-**
	ST	**-**	**-**	**-**	**-**	** + **	**-**	**-**	**-**	**-**	** + **	**-**	** + **	**-**	** + **	** + **	**-**	**-**	**-**	**-**	** + **	** + **	**-**	** + **	**-**	**-**
*Indigofera trita* L. F	AD	**-**	**-**	** + **	** + **	**-**	**-**	**-**	**-**		**-**	**-**	** + **	**-**	**-**	**-**	**-**	**-**	**-**	**-**	**-**	** + **	**-**	**-**		**-**
	AB	**-**	**-**	** + **	** + **	**-**	** + **	**-**	** + **	** + **	** + **	**-**	** + **	**-**	** + **	**-**	**-**	**-**	**-**	**-**	**-**	** + **	**-**	** + **	** + **	**-**
	ST	**-**	**-**	** + **	** + **	** + **	**-**	**-**	**-**	**-**	** + **	** + **	** + **	** + **	** + **	** + **	** + **	**-**	**-**	**-**	** + **	** + **	** + **	** + **	** + **	**-**

Abbreviations: (AB): abaxial leaf, (AD): adaxial leaf, (ST): stem, (S): subtype, (Peri): pericytic, (Desmo): desmosocytic, ( Hemipara): hemiparacytic, (Para): paracytic, (Dia): diacytic, (ITBPD): intermediate type between paracytic and diacytic, ( Polo): polocytic, (Aniso): anisocytic, (Amphianiso): amphianisocytic, (Anisotri): anisotricytic, (Amphianisotri): amphianisotricytic, (Isotri): iasotricytic, (Latero): laterocytic,(Stauro): staurocytic, (Tetra): tetracytic, (Cyclo): cyclocytic, (Anomo): anomocytic, (Stephano): stephanocytic,(Hexa): hexacytic, ( +): present, (-): absent

**Table 5:** Organographic distribution of various abnormal stomatal types in the studied taxa of *Indigofera*

Taxa/ Character	AD/AB/ST	Abnormal stomata																
		Contiguous stomata				Twin stomata				Triple stomata	Single guard cell			Giant stomata		Arrested stomata		Stoma associated with foot cell
		Sub-I	Sub-II	Sub-III	Sub-IV	Sub-I	Sub-II	Sub-III	Sub-IV		Sub-I	Sub-II	Sub-III	Sub-I	Sub-II	Sub-I	Sub-II	
*Indigofera arabica* Jaub. & Spach	AD	** + **	** + **	**-**	**-**	**-**	**-**	**-**	** + **	**-**	**-**	**-**	**-**	**-**	** + **	**-**	** + **	**-**
	AB	** + **	** + **	**-**	**-**	**-**	**-**	**-**	**-**	**-**	**-**	**-**	**-**	**-**	** + **	**-**	** + **	** + **
	ST	**-**	**-**	**-**	**-**	**-**	**-**	**-**	**-**	**-**	**-**	**-**	**-**	**-**	**-**	**-**	**-**	**-**
*Indigofera argentea* Burm.f	AD	** + **	** + **	** + **	**-**	**-**	**-**	**-**	**-**	**-**	**-**	**-**	**-**	**-**	**-**	**-**	**-**	**-**
	AB	** + **	**-**	**-**	**-**	**-**	**-**	**-**	**-**	**-**	**-**	**-**	**-**	**-**	**-**	**-**	**-**	**-**
	ST	**-**	**-**	**-**	**-**	**-**	**-**	**-**	**-**	**-**	**-**	**-**	**-**	**-**	**-**	**-**	**-**	**-**
*Indigofera articulata* Gouan	AD	** + **	** + **	**-**	**-**	** + **	**-**	**-**	** + **	**-**	**-**	**-**	**-**	**-**	**-**	**-**	**-**	**-**
	AB	** + **	** + **	**-**	**-**	**-**	**-**	**-**	**-**	**-**	**-**	**-**	**-**	**-**	**-**	**-**	** + **	**-**
	ST	**-**	**-**	**-**	**-**	**-**	**-**	** + **	**-**	**-**	**-**	**-**	**-**	** + **	**-**	**-**	**-**	**-**
*Indigofera coerulea* var. *coerulea* Roxb	AD	** + **	** + **	**-**	**-**	**-**	**-**	**-**	** + **	**-**	**-**	**-**	**-**	**-**	**-**	**-**	**-**	** + **
	AB	** + **	** + **	**-**	**-**	**-**	**-**	**-**	**-**	**-**	**-**	**-**	**-**	**-**	**-**	**-**	**-**	**-**
	ST	**-**	**-**	**-**	**-**	**-**	**-**	**-**	**-**	**-**	**-**	**-**	**-**	**-**	**-**	**-**	** + **	**-**
*Indigofera colutea* (Burm.f.) Merr	AD	** + **	**-**	**-**	**-**	**-**	**-**	**-**	**-**	**-**	**-**	** + **	**-**	**-**	**-**	**-**	** + **	**-**
	AB	** + **	** + **	**-**	**-**	**-**	**-**	**-**	**-**	**-**	**-**	**-**	**-**	**-**	**-**	**-**	**-**	**-**
	ST	**-**	**-**	**-**	**-**	**-**	**-**	**-**	**-**	**-**	**-**	**-**	**-**	** + **	**-**	**-**	**-**	**-**
*Indigofera cordifolia* B. Heyne ex Roth	AD	** + **	**-**	**-**	**-**	**-**	** + **	**-**	**-**	**-**	**-**	**-**	**-**	**-**	**-**	**-**	**-**	**-**
	AB	** + **	** + **	** + **	**-**	**-**	**-**	**-**	**-**	**-**	**-**	**-**	**-**	**-**	**-**	** + **	**-**	**-**
	ST	**-**	**-**	**-**	**-**	**-**	**-**	**-**	**-**	**-**	**-**	**-**	**-**	**-**	**-**	** + **	**-**	**-**
*Indigofera hochstetteri* Baker	AD	** + **	** + **	** + **	** + **	** + **	** + **	**-**	** + **	**-**	**-**	**-**	** + **	**-**	**-**	**-**	**-**	**-**
	AB	** + **	** + **	** + **	**-**	**-**	**-**	**-**	** + **	**-**	**-**	**-**	**-**	**-**	** + **	**-**	** + **	**-**
	ST	**-**	**-**	**-**	**-**	**-**	**-**	**-**	**-**	**-**	**-**	**-**	**-**	**-**	**-**	**-**	**-**	**-**
*Indigofera oblongifolia* Forssk	AD	** + **	** + **	**-**	**-**	**-**	**-**	**-**	** + **	**-**	**-**	**-**	**-**	**-**	**-**	**-**	** + **	**-**
	AB	** + **	**-**	**-**	**-**	** + **	**-**	**-**	**-**	**-**	**-**	**-**	**-**	**-**	**-**	** + **	**-**	**-**
	ST	**-**	**-**	**-**	**-**	**-**	**-**	**-**	**-**	**-**	**-**	**-**	**-**	** + **	**-**	**-**	**-**	**-**
*Indigofera sessiliflora* DC	AD	** + **	** + **	** + **	**-**	**-**	**-**	**-**	**-**	**-**	**-**	**-**		**-**	**-**	**-**	**-**	**-**
	AB	** + **	** + **	** + **	**-**	** + **	**-**	**-**	**-**	**-**	**-**	**-**	** + **	**-**	**-**	**-**	**-**	**-**
	ST	**-**	**-**	**-**	**-**	**-**	**-**	**-**	**-**	**-**	**-**	**-**		**-**	**-**	**-**	**-**	**-**
*Indigofera spiniflora* Hochst. ex Boiss	AD	** + **	** + **	** + **	**-**	**-**	**-**	**-**	**-**	**-**	**-**	**-**	**-**	**-**	**-**	**-**	**-**	**-**
	AB	** + **	**-**	**-**	**-**	** + **	**-**	**-**	**-**	**-**	**-**	**-**	**-**	**-**	**-**	**-**	**-**	**-**
	ST	**-**	**-**	**-**			**-**	**-**	**-**	**-**	**-**	**-**	**-**	**-**	**-**	**-**	**-**	**-**
*Indigofera spinosa* Forssk	AD	** + **	** + **	**-**	**-**	** + **	**-**	**-**	**-**	**-**	** + **	** + **	** + **	**-**	**-**	**-**	**-**	**-**
	AB	** + **	** + **	**-**	**-**	**-**	**-**	**-**	**-**	**-**	**-**	**-**	**-**	**-**	**-**	**-**	**-**	**-**
	ST	**-**	**-**	**-**	**-**	**-**	**-**	**-**	**-**	**-**	**-**	**-**	**-**	** + **	**-**	**-**	**-**	**-**
*Indigofera subulata* Vahl ex Poir. var.* subulata*	AD	** + **	** + **	**-**	**-**	**-**	**-**	**-**	**-**	**-**	**-**	**-**	**-**	**-**	**-**	**-**	**-**	**-**
	AB	** + **	**-**	**-**	**-**	**-**	**-**	**-**	**-**	**-**	**-**	**-**	**-**	**-**	**-**	**-**	**-**	**-**
	ST	**-**	**-**	**-**	**-**	**-**	**-**	**-**	**-**	**-**	**-**	**-**	**-**	**-**	**-**	**-**	**-**	**-**
*Indigofera trita* L. F	AD	** + **	** + **	** + **	**-**	**-**	**-**	**-**	** + **		**-**	**-**	** + **	**-**	** + **	**-**	**-**	**-**
	AB	** + **	** + **	** + **	**-**	** + **	** + **	** + **	** + **	** + **	**-**	** + **	**-**	**-**	**-**	**-**	**-**	** + **
	ST	** + **	** + **	** + **	**-**	** + **	**-**	**-**	**-**	**-**	**-**	**-**	**-**	**-**	**-**	**-**	**-**	**-**

Abbreviations: (AB): abaxial leaf, (AD): adaxial leaf, (ST): stem, (Sub): subtype, ( +): present, (-): absent

### Micro-morphological characteristics of epidermal cells

Micro-morphological characteristics of the foliar and stem epidermal cells showed a great diversity among studied taxa. The most critical aspects of the diversity of epidermal cells were the shape, frequency, wall thickness, ornamentation of the cuticle, and occurrence of crystals.

Light microscopy and SEM observations of the adaxial and abaxial leaf surfaces revealed that the shape of the epidermal cells was tetragonal, pentagonal, and hexagonal, with straight to slightly undulate anticlinal walls in most of the studied taxa (Figs. 1, 2, 3, 4, 5, 6 and Table 2). In addition, polygonal epidermal cells were observed in the leaves of all taxa except the abaxial surface of *I. spinosa* Forssk. Moreover, irregular epidermal cells were present in all taxa except *I. coerulea* var. *coerulea* Roxb. and *I. oblongifolia*. Generally, the cells along the veins of the leaf lamina were elongated. The anticlinal wall patterns were predominantly undulate to deeply undulate in *I. spinosa* and *I. subulata* Vahl ex Poir. var. *subulata*, while straight to slightly arched in *I. oblongifolia* and slightly arched to arched in the abaxial surface of *I. coerulea* var. *coerulea* (Figs. 1, 2, 3, 4, 5, 6 and Table 2).

The anticlinal walls were commonly smooth in most studied taxa (Figs. 1, 2, 3, 4, 5, 6 and Table 2). However, it varied from smooth to beaded in *I. sessiliflora*, *I. cordifolia* B. Heyne ex Roth, and *I. spiniflora* Hochst. ex Boiss., the abaxial and adaxial surfaces of *I. colutea* and *I. spinosa*, the abaxial surface of *I. hochstetteri* and the stems of *I. argentea*, *I. trita*, *I. subulata* var. *subulata*, and *I. hochstetteri*. Smooth to ridged walls were observed on the abaxial and adaxial surfaces of *I. argentea* and the adaxial surface of *I. hochstetteri*. In contrast, ridges to knobes were recorded on the abaxial and adaxial surfaces of *I. subulata* var. *subulata*. Moreover, irregularly thickened walls were observed on the abaxial and adaxial surfaces of *I. sessiliflora*, the adaxial surface of *I. cordifolia*, the abaxial surface of *I. spiniflora*, and the stem of *I. oblongifolia* (Nos. 1–5 in Figs. 1 and 2).

Most taxa exhibited a rugulate ornamentation of the cuticle surface (homogenous) with lattice-like extrusions lumen. Meanwhile, *I. hochstetteri*, the adaxial surfaces of *I. sessiliflora*, *I. trita*, *I. spiniflora*, *I. argentea*, and the abaxial surface *of I. spinosa* had a ruminate leaf cuticle. The abaxial surface of *I. colutea*, the adaxial surface of I. spinosa, and *I. oblongifolia* had a rugulate to ruminate cuticle surface. In contrast, both leaf surfaces of *I. cordifolia*, *I. subulata* var. *subulata*, and the abaxial surface of *I. argentea* had a reticulate cuticle surface. Calcium oxalate crystals were observed in a few taxa. For example, raphides were observed on both surfaces of *I. arabica* Jaub. & Spach and solitary crystals were found on the abaxial surfaces of *I. trita*.

### Micro-morphological characteristics of mature stomata

Micro-morphological characteristics of the foliar and stem stomatal cells showed a great diversity among the taxa under investigation. The most critical aspects of the diversity of stomatal cells were the shape, position, frequency, stomatal index, and stomatal types.

### Stomatal shape and position

Most studied taxa had circular, oblong, and elliptic stomata to wide elliptic shapes. Only *I. cordifolia* showed a narrow elliptic shape of stomata (Fig. 5 and Table 2). The leaves of most taxa had stomata situated slightly sunken to sunken in both their adaxial and the abaxial surfaces (Figs. 5 and 6 a, b and Table 2). Only *I. trita* leaf surfaces and the abaxial surface of *I. coerulea* var. *coerulea* and *I. spiniflora* had stomata at the same level as the epidermal cells (Fig. 6: 12a, b; Table 2). In contrast, the stems of most taxa had stomata positioned at the same level with epidermal cells (Figs. 5c and 6c and Table 2). Only *I.argentea* and *I. spinosa* stems had slightly sunken stomata (Figs. 5: 2c, 6: 11c and Table 2).

### Stomatal cell frequency and stomatal index

Both stomatal cell frequency and stomatal index showed a great diversity among the studied taxa. The stomatal cell frequency was highest on the adaxial surface of *I. hochstetteri,* with an average of 38.67, while it was lowest on the adaxial surface of *I. spinosa,* with an average of 9.37 (Table 3). The highest value of the stomatal index was observed in the adaxial surface of *I. hochstetteri* (27.46%), whereas the lowest value was observed in the abaxial surface of *I. oblongifolia. (*9.95%) (Table 3).

### Stomatal types

Various types of stomata were observed on both leaf and stem surfaces. The leaves of all studied taxa were amphistomatic. Stomatal structures were acyclic, monocyclic, or amphicyclic. The mature stomata generally exhibited prominent T-shaped cuticular thickening at both poles. We classified the stomata according to the occurrence, number, orientation, and size of subsidiary cells into two main types: normal and abnormal (Figs. 1, 2, 3, 4, 5 and 6).

#### A- Normal stomata

Light microscopy and SEM observations of the leaf and stem surfaces revealed the occurrence of 19 normal stomatal types with nine subtypes, as follows:

1. **Pericytic:** This stoma type is characterized by having one subsidiary cell (distinct or indistinct) surrounding the stoma in a free position, without any linkage with the subsidiary cell wall (Dilcher 1974; Badry 2018) (No. 6 in Figs. 1 and 2).
2. **Desmocytic:** This stoma type contains a single distinct or indistinct subsidiary cell surrounding the stoma with a conjoint anticlinal cell wall between the pole of guard cells and the subsidiary cell wall (Dilcher 1974; Badry 2018) (No. 7 in Figs. 1 and 2).
3. **Hemiparacytic:** This stoma type comprises a single subsidiary cell laying parallel to one guard cell and a long axis of the pore from one side (Fryns-Claessens and Van Cotthem 1973; Dilcher 1974; Badry 2018; Nisa et al. 2019). Two subtypes of hemiparacytic were recognized:
  **Subtype-I:** In this subtype, one subsidiary cell is adjacent to one guard cell, enclosing it and parallel to its long axis, and the other guard cell has two normal epidermal cells surrounding it, with (1 + 2) arrangement (No. 8 in Figs. 1 and 2).
  **Subtype-II:** In this subtype, one subsidiary cell is adjacent to one guard cell, enclosing it and parallel to its long axis, and the other guard cell has three normal epidermal cells surrounding it, with (1 + 3) arrangement (No. 9 in Figs. 1 and 2).
4. **Paracytic:** This stoma type consists of two subsidiary cells surrounding the stoma and is oriented parallel to the long axis of the pore and the guard cells, with variations in shape and size (Dilcher 1974; Badry 2018; Grohar et al. 2022). Two subtypes of paracytic stomata were recognized:
  **Subtype-I "equal paracytic":** In this subtype, two equal subsidiary cells surround the guard cells with conjoint walls on both poles (No. 10 in Figs. 1 and 2).
  **Subtype-II "unequal paracytic":** In this subtype, two unequal subsidiary cells surround the guard cells with conjoint walls on both poles (No. 11 in Figs. 1 and 2).
5. **Diacytic:** A stoma is surrounded by two subsidiary cells with a standard wall perpendicular to the guard cells (i.e., linked laterally to the guard cells) (Dilcher 1974; Inamdar and Patel 1976). The subsidiary cells may vary in size and shape (No. 12 in Figs. 1 and 2).
6. **Intermediate type between paracytic and diacytic:** This stoma type is a transitional case between paracytic and diacytic. In this type, the two subsidiary cells surround the stoma in oblique orientation instead of the parallel or at proper angle orientation (Badry 2018; Inamdar & Patel 1976) (No. 13 in Figs. 1 and 2).
7. **Polocytic:** This stoma type is characterized by two distinct or indistinct subsidiary cells that surround the stoma, and the guard cell's distal end is connected to the distal polar side of a single subsidiary cell. The link is marginopolar and never below the midway between the guard cells. The subsidiary cell has a U-shape (Dilcher 1974) (No. 14 in Figs. 1 and 2).
8. **Anisocytic:** This stoma type consists of three subsidiary cells surrounding the stoma, with variations in shape and position, and one of them is significantly smaller than the other two (Metcalfe and Chalk 1950; Dilcher 1974; Baranova 1992; Badry 2018; Nisa et al. 2019) (No. 15 in Figs. 1 and 2).
9. **Amphianisocytic:** This stoma type contains multiple subsidiary cells surrounding the stoma as double rings, with the inner one consisting of three cells (two large, one smaller) and the outer one, which may be incomplete, consisting of 2˗3 or 4 cells (Dilcher 1974; Nisa et al. 2019) (No. 16 in Figs. 1 and 2).
10. **Anisotricytic:** This stoma type comprises three subsidiary cells surrounding the stoma, with variations in shape and position; one is significantly larger than the other two (Badry 2018) (No. 17 in Figs. 1 and 2).
11. **Amphianisotricytic:** This stoma type consists of double layers of subsidiary cells surrounding guard cells, with the inner layer consisting of 3 cells (one large, two smaller); the outer layer may be incomplete and composed of 2˗3 or 4 cells (Baranova 1992) (No. 18 in Figs. 1 and 2).
12. **Isotricytic:** This stoma type is recognized by having three subsidiary cells surround the stoma, and they are more or less equal in size, with variation in shape and position (Dilcher 1974; Prabhakar 2004; Nisa et al. 2019) (No. 19 in Figs. 1 and 2).
13. **Laterocytic:** The stoma is accompanied by three or more lateral subsidiary cells bordering both sides of the guard cells, and the anticlinal walls separate the adjacent subsidiary cells (Baranova 1983). Five arrangements were recognized in addition to the typical form:
  **Subtype-I:** In this subtype, the stoma is surrounded by three subsidiary cells; one is parallel to one side of the guard cells, and the two other cells are on the other side of the guard cells with a (1 + 2) arrangement (No. 20 in Figs. 1 and 2).
  **Subtype-II:** In this subtype, the stoma is surrounded by four subsidiary cells, one parallel to one side of the guard cells and the other three cells on the other side with (1 + 3) arrangement (No. 21 in Figs. 1 and 2).
  **Subtype-III:** In this subtype, the stoma is surrounded by four subsidiary cells, each of two on either side of the guard cells with a (2 + 2) arrangement (No. 22 in Figs. 1 and 2).
  **Subtype-IV:** In this subtype, the stoma is surrounded by five subsidiary cells; two are situated on one side of the guard cells, and three are on the other, and has a (2 + 3) arrangement (No. 23 in Figs. 1 and 2).
  **Subtype-V:** In this subtype, the stoma is surrounded by six subsidiary cells; three are situated on one side of the guard cells, and three are on the other side, with (3 + 3) arrangement (No. 24 in Figs. 1 and 2).
14. **Staurocytic:** This stoma type comprises four subsidiary cells surrounding the stoma, and they are variable in size and shape, and their radial conjoint walls are connected crosswise to the guard cells (i.e., two walls are polar, and the other two are lateral) (Dilcher 1974; Badry 2018) (No. 25 in Figs. 1 and 2).
15. **Tetracytic:** This stoma type contains four subsidiary cells surrounding the stoma, variable in shape and size; two are polar, while the others are lateral (Dilcher 1974) (No. 26 in Figs. 1 and 2).
16. **Cyclocytic:** The stoma of this type is surrounded by a narrow ring of four or more small subsidiary cells, and they are variable in position, size, and shape (Gill and Karatela 1983) (No. 27 in Figs. 1 and 2).
17. **Anomocytic:** The stoma of this type is surrounded by four or more subsidiary cells indistinguishable from the remainder of the epidermal cells, and they are variable in size, shape, and position (varies from tetracytic and staurocytic types) (Metcalfe and Chalk 1950) (No. 28 in Figs. 1 and 2).
18. **Stephanocytic:** The stoma of this type is surrounded by a well-defined rosette of four or more radiating subsidiary cells, and they are variable in position, shape, and size (Loockerman and Jansen 1996) (No. 29 in Figs. 1 and 2).
19. **Hexacytic:** This stoma type consists of six subsidiary cells surrounding the stoma; four are adjacent to the guard cells on the lateral sides parallel to the long axis of the guard cells, and the other two are situated on the polar sides. These can be distinguished from the epidermal cells (Tomlinson 1969) (No. 30 in Figs. 1 and 2).

#### B- Abnormal stomata

Light microscopy and SEM observations of the leaf and stem surfaces revealed the occurrence of seven abnormal stomatal types with 13 subtypes, as follows:

1. **Contiguous stomata:** This type of stomata comprises two stomata that lay adjacent and share one common subsidiary cell or more (Inamdar and Patel 1976; Mandal et al. 2012). Four arrangements were recognized in addition to the typical form:
  **Subtype-I:** In this subtype, two stomata lay adjacent and share one common subsidiary cell. The contributed stomata might be of the same or different types (No. 1 in Figs. 3 and 4).
  **Subtype-II:** In this subtype, two stomata lay adjacent and share two common subsidiary cells. The contributed stomata might be of the same or different types (No. 2 in Figs. 3 and 4).
  **Subtype-III:** In this subtype, three stomata lay adjacent and share one common subsidiary cell. The contributed stomata might be of the same or different types (No. 3 in Figs. 3 and 4).
  **Subtype-IV:** In this subtype, four stomata lay adjacent and share one common subsidiary cell. The contributing stomata might be of the same or different types (No. 4 in Figs. 3 and 4).

2. **Twin stomata:** This type of stomata is characterized by the two stomata being developed from two adjacent meristemoids (Inamdar and Patel 1976; Mandal et al. 2012). Four arrangements were recognized based on the orientation of the two stomata forming the twin stomata (or on the plane of division in the adjacent meristemoids):
  **Subtype-I (Juxtaposed and parallel twin stoma)**: In this subtype, the two stomata are positioned parallel to each other (side-by-side) (No. 5 in Figs. 3 and 4).
  **Subtype-II (Perpendicular):** In this subtype, one of the two stomata is positioned perpendicular to the second one (No. 6 in Figs. 3 and 4).
  **Subtype-III (Superimposed):** In this type, two stomata lay in a single line, touching pole to pole (Nos. 7, 8 in Figs. 3 and 4).
  **Subtype-IV (Twin stomata associated with a contiguous stoma):** In this subtype, obliquely oriented twin stomata are connected to another stoma as a contiguous type through a cytoplasmic bridge (Nos. 9, 10 in Fig. 3; No. 9 in Fig. 4).

3. **Triple stomata:** This type of stomata is developed from three adjacent meristemoids with three pores and three guard cells (No. 11 in Fig. 3; No. 10 in Fig. 4).
4. **Single guard cell:** This type of stomata has only one guard cell with or without a pore (Patel and Inamdar 1971; Mandal et al. 2012). We recognized three subtypes as follows:
  **Subtype-I:** In this subtype, the single guard cell contains a pore (No. 12 in Fig. 3; No. 11 in Fig. 4).
  **Subtype-II:** In this subtype, the single guard cell contains no pore (No. 13 in Fig. 3; No. 12 in Fig. 4).
  **Subtype-III:** In this subtype, the single guard cell with or without a pore is juxtaposed and attached to a normal stoma (Nos. 14, 15 in Fig. 3; No. 13 in Fig. 4).

5. **Giant stomata:** These are significantly larger (15–20%) than most others. Usually, there are radiating striations on the subsidiary cells (Mandal et al. 2012) (No. 16 in Fig. 3; No. 14 in Fig. 4). Another arrangement was recognized in addition to the typical form.
  **Subtype-I:** In this subtype, the Giant stoma is always associated with a contiguous stoma (No. 17 in Fig. 3; Nos. 15, 16 in Fig. 4).

6. **Arrested stoma:** In this type, the stomatal ontogeny is stopped at an earlier development stage. The stomata development is terminated at the meristemoid stage, the guard mother cell stage, or been cut off from 1, 2, or 3 subsidiary cells (Inamdar et al. 1971) (No. 18 in Fig. 3; No. 17 in Fig. 4). Another arrangement was recognized besides the typical form.
  **Subtype-I:** In this subtype, the Arrested stoma is always associated with a contiguous stoma (No. 19 in Fig. 3; Nos. 18, 19 in Fig. 4).

7. **Stoma associated with trichome foot cell:** This stoma type is characterized by the stomatal guard cells juxtaposed attached with the foot cell of a trichome (No. 20 in Figs. 3 and 4).

Based on the occurrence of different types of abnormal stomata, we designed the following artificial key to differentiate the taxa under investigation.

- 1a. Contiguous and Twin types of stomata are present ………………………………………………………… 2
- 1b. Other types of stomata are present ……………………………………….………………………………… 4
- 2a. Twin type of stomata is present only on abaxial leaf ………………………………………….….*I. spiniflora*
- 2b. Only Contiguous type of stomata is present ………………………………..………………..………….… 3
- 3a. Two subtypes (I, II) of Contiguous stomata are present ……………………….….. *I. subulata* var.* subulata*
- 3b. Three subtypes (I, II, III) of Contiguous stomata are present ……………………….……..……. *I. argentea*
- 4a. Triple type of stomata is present on abaxial leaf ……………..……………………….……………… *I. trita*
- 4b. Triple type of stomata is absent ……………………………….…………………………………………… 5
- 5a. Stomata is associated with foot cell ……………………………….……………………………….………. 6
- 5b. Stomata is not associated with foot cell ……………………………..……………………………..………. 7
- 6a. Giant type of stomata is present on the abaxial and adaxial of the leaf …………………………… *I. arabica*
- 6b. Giant type of stomata is absent …………………………..……..………….………. *I. coerulea* var. *coerulea*
- 7a. Twin type of stomata is absent ……………………………………………………………….…….……… 8
- 7b. Twin type of stomata is present …………………………………….…………………………………..… 9
- 8a. Arrested type of stomata is present on the abaxial of leaf and stem …………………………… *I. cordifolia*
- 8b. Arrested type of stomata is absent ……..…………………………………………………………. *I. colutea*
- 9a. Giant and Arrested types of stomata are absent ……………………………………………..……………. 10
- 9b. Giant and Arrested types of stomata are present …..…………………………………………..…………… 11
- 10a. Subtypes I and II of single guard cell stomata are present on adaxial leaf.……………………. *I. spinosa*
- 10b. Subtypes III only of single guard cell stomata is present on abaxial leaf …………………….. *I. sessiliflora*
- 11a. All four subtypes of Contiguous stomata are present …………………………….….……… *I. hochstetteri*
- 11b. Two subtypes (I &II) only of Contiguous stomata are present ………………………….…….………… 12
- 12a. Three subtypes (I, III & IV) of Twin stomata are present ….……………………….…..……. *I. articulata*
- 12b. Two subtypes (I & IV) of Twin stomata are present …….…………………..…..………… *I. oblongifolia*

## Discussion

From the biosystematic point of view, stomatal complexes are one of the diagnostic characteristics of different plant taxa. Since they are usually stable among other environmental conditions, various studies have utilized these characteristics in delimiting plant groups, eliminating the controversy of those groups, and even interpreting the phylogeny of the groups (Stace 1965b; Payne 1970; Baranova 1972; Dilcher 1974; Gill et al. 1982; Carpenter 2005). Moreover, the taxonomic significance of the foliar epidermis has been emphasized for many plant groups (Pant and Banerji 1965; Payne 1970; Gopal and Shah 1970; Dehgan 1980; Rao and Ramayya 1982; Gill et al. 1982; Nyawuame and Gill 1990; Gill and Nyawuame 1990; Idu et al. 2000; Adedeji and Dloh 2004; Abdulrahaman and Oladele 2005; Shaheen et al. 2009a, b, c; Van Cotthem 1970; Ghahremaninejad et al. 2012; Albert and Sharma 2013; Thakur and Patil 2014; Patil et al. 2015; Grohar et al. 2022). However, few studies have shed light on the taxonomic importance of the stem epidermal features (e.g., Inamdar & Chohan 1969a,b; Rao & Ramayya 1982; Rao 1991).

Many researchers have indicated that stomatal and epidermal cell characteristics affect the drought resistance and water use efficiency of plants. The modifications of stomatal and epidermal cell characteristics in response to drought may vary depending on plant species and plant varieties (Pirasteh-Anosheh et al. 2016). A great number of studies have indicated that plant adaptation to drought stress conditions may include increasing of stomatal density (Xu and Zhou 2008) and/or decreasing of stomatal size (Martínez et al. 2007). Xu and Zhou (2008) have found a positive and significant correlation between stomatal density with stomatal conductance, CO_2_ level, and water use efficiency. In this study, we described the quantitative and qualitative characteristics of leaf and stem epidermal cells and stomata of 13 taxa of *Indigofera*. The attributes of epidermal cells and stomata on leaf surfaces vary from taxon to taxon and in the same taxon (Vijay-Kumar and Ramayya 1987; Quesada 1997). The investigated taxa exhibited various types of mature stomata on the same leaf surface. Differences in mature stomatal types arise from cell shape and polarity variations during developmental stages (Rudall 2023). This stomatal diversity has been previously reported in Fabaceae (Solereder 1908; Metcalfe and Chalk 1950; Shah and Gopal 1969a, b; Grohar et al. 2022) and in the genus *Indigofera* (Gill et al. 1982; Vijay-Kumar and Ramayya 1987; Quesada 1997). Moreover, Metcalfe & Chalk (1979) emphasized that the presence and combination of different types of stomata on the epidermal surfaces can be helpful in the classification and delimitation of the taxa they studied.

The study revealed significant diversity in the stomatal characteristics of the studied taxa. Leaves of all the studied taxa were observed to be amphistomatic (Metcalfe and Chalk 1950; Vijay-Kumar and Ramayya 1987; Quesada 1997). Among the analyzed taxa, 19 normal stomata (with nine subtypes) and seven abnormal stomatal complexes (with 13 subtypes) were recognized. Abnormal stomatal patterns are usually much different from normal (Payne 1970). Metcalfe & Chalk (1979) admitted stomatal abnormalities in more than 60 monocots, dicots, and gymnosperms species. Several authors have recorded stomatal abnormalities in different genera of the family Fabaceae (Farooqui et al. 1989; Gan et al. 2010; Albert and Sharma 2013). However, a few researchers have recorded stomatal abnormalities in the genus *Indigofera* (Quesada 1997).

The diversity of stomatal types is correlated with the vast variations in the count, position, and shape of subsidiary and epidermal cells. Since most plants respond to drought stress by closing their stomata, subsidiary cells contribute with guard cells to perform the closure by providing a mechanical advantage that facilitates guard cell movements and by serving as a reservoir for ions and water (Gray et al. 2020). Vijay-Kumar and Ramayya (1987) reported anisocytic stomata as the predominant stomatal complex in the genus *Indigofera*, in addition to a few anomocytic and tetracytic and rarely paracytic stomata. Later, in his study on seven Cuban taxa of *Indigofera*, Quesada (1997) reported anisocytic, anomocytic, tetracytic, paracytic, hemiparacytic, brachyparacytic, actinocytic, ciclocytic, and diacytic stomata in addition to five types of abnormal stomata (adjacent stomata with a common subsidiary cell, giant stomata, juxtaposed stomata, stomata with only one guard cell, and superposed stomata). In the present study, some stomatal types are newly recorded and described, especially some abnormal ones. Thus, this study represents a new addition to the micro-morphological features of the genus *Indigofera*. The studied taxa of *Indigofera* can be delimited based on the unique size, frequency, and stomatal types.

The occurrence, number, and orientation of stomata are mainly relying on the successive events of stomatal development. First, a protodermal cell turns into a meristemoid mother cell, which, in turn, goes an asymmetric entry division to produce a small triangular cell (meristemoid) and a larger cell (stomatal-lineage ground cell). The large cell can differentiate into a pavement cell of the epidermis or alternatively initiate an asymmetric spacing division to produce another meristemoid that is always oriented away from the existing meristemoid. Meristemoid divisions are called amplifying divisions and can occur up to three or four times (Serna 2009; Pillitteri and Dong 2013). These divisions aim to regenerate a meristemoid and increase the total number of stomatal lineage ground cells produced by a single lineage. After a variable number of amplifying divisions, the meristemoid cells lose their stem cell activity and develop into oval-shaped guard mother cells. Finally, a guard mother cell divides once symmetrically to produce two guard cells, which do not divide further (Bergmann and Sack 2007; Pillitteri and Dong 2013). The outcome of all these divisions is an increase in the number of stomata and pavement cells.

The number of stomata produced in the leaf relies on the frequency of occurrence of the different types of asymmetric divisions (different cycles of entry and amplifying divisions). Thus, the observed differences in stomata number and orientation among different parts of a plant and among different taxa may have resulted from the variations of these different types of asymmetric divisions (Geisler and Sack 2002; Bergmann and Sack 2007; Rudall 2023). In the model plant *Arabidopsis thaliana*, several genes were reported to regulate the divisions in the stomatal lineage and, subsequently, the stomata's number, distribution, and orientation. For example, mutations in *TOO MANY MOUTHS* (*TMM*) and *FOUR LIPS* (*FLP*) genes result in stomatal clustering and increased precursor cell formation (Yang and Sack 1995). Also, a mutation in the *STOMATAL DENSITY AND DISTRIBUTION 1* (*SDD1*) gene disrupted the establishment of the stomatal pattern andresulted in stomata clustering with two- to four-fold stomatal density (Von Groll et al. 2002). The overexpression of *SDD1*in the leaves of wild-type *Arabidopsis* resulted in an opposite phenotype with a two to three-fold decrease in stomatal density and the formation of arrested stomata. The spacing cell between the two adjacent stomata was missing in all these mutants. We reported similar patterns of stomata, including contiguous stomata (two stomata lay adjacent and share one common subsidiary cell or more), Twin stomata (two stomata developed from two adjacent meristemoids), and arrested stomata (stomatal ontogeny is stopped at earlier development stage).

The proper functioning of stomata is directly linked to its shape, arrangement pattern, and frequency. It is not only genetically controlled but also affected by several environmental factors, including light, temperature, carbon dioxide, nutrient and water availability, internal architecture, and insertion level on leaves (raised, at the same level, or sunken) (Croxdale 2000; Liu et al 2023). Within *Indigofera*, the shape of stomatal complexes is considered of great diagnostic importance. It is generally circular, oblong, and elliptic to widely elliptic with rounded to retuse ends in most of the studied taxa, while narrowly elliptic in the leaves of *I. cordifolia.* Likewise, stomatal structures in leaves and stems were generally acyclic, hemicyclic, and monocyclic; however, amphicyclic (dicyclic) stomatal arrangement was observed only on the stems of some studied taxa (*I. arabica*, *I. sessiliflora*, *I. trita*; and *I. hochstetteri*) (Figs. 1, 2, 3, 4 and Table 3). Stomatal orientation (insertion level/ position) may be of taxonomic importance in addition to its physiological role. Dörken et al. (2020) reported that xerophytes inhabiting arid environments, resembling the desert habitats of the studied taxa, exhibited sunken stomata on their photosynthetic organs. Sunken stomata contributed less to the total leaf conductance for water than superficial stomata in dicot species (Šantrůček 2022). Moreover, Koster and Baas (1982) have suggested that sunken stomata might participate in plant protection from herbivores. Our results indicated that the most common foliar stomatal orientation observed was slightly sunken to sunken. In comparison, both leaf surfaces of *I. trita* and the abaxial surface of *I. coerulea* var. *coerulea* and *I. spiniflora* have stomata at the same level (Figs. 5 and 6 a, b and Table 2). However, in the stem, most stomata were oriented at the same level of the epidermis, except in the stem of *I. argentea* and *I. spinosa*, which were slightly sunken (Figs. 5 and 6 c and Table 2).

Stomatal morphological characteristics such as size, frequency, and distribution may affect the function of stomata quite remarkably (Croxdale 2000; Liu et al 2023). Stomatal size variation can be influenced by various factors, including genetic characteristics and environmental factors such as light and water (James and Bell 2000; Silva et al. 2009; Fanourakis et al. 2019). The foliar guard cell size, stomatal frequency, and stomatal index provide quantitative values that serve as parameters for comparisons among taxa (Adedeji et al. 2007; Essiett and Iwok 2014). The size of guard cells and stomatal pores showed a remarkable variation among the studied taxa. The smallest and narrowest stomata were recorded on the adaxial surface of *I. cordifolia* (13.80 × 10.35 µm). The abaxial surface of *I. spinosa* had the longest stomata (27.63 µm), whereas the abaxial surface of *I. trita* had the widest stomata (23.72 µm). The smallest and narrowest stomatal pores were recorded on the adaxial surface of *I. cordifolia* (5.26 × 1.02 µm), while the longest and widest stomatal pores were recorded on the abaxial surface of *I. spinosa* (15.91 × 2.25 µm). Several studies have documented the negative relationship between stomatal frequency and stomatal size (Franks & Beerling 2009). Stomatal cell frequency showed a great diversity among the taxa. The adaxial surface of *I. hochstetteri* had the highest stomatal count per unit area, with an average epidermal cell frequency of 38.67. Meanwhile, the lowest stomatal cell frequency was observed on the adaxial surface of *I. spinosa*, with an average epidermal cell frequency of 9.37. Engineer et al. (2014) reported that *Arabidopsis* adjusts the stomatal frequency in response to CO_2_ level by mediating the peptide *EPIDERMAL PATTERNING FACTOR 2* (*EPF2*).

The stomatal index is highly constant for a particular species and can be used for taxa delimitation (Abdulrahaman and Oladele 2003, 2005; Adedeji et al. 2007). Within the studied taxa, the stomatal index on the abaxial leaf surface had a low value compared to the adaxial surface of most of the studied taxa except for *I. argentea* and *I. articulata.* This finding contradicts the findings of Tripathi and Mondal (2012). On the other hand, stomatal indexes in the abaxial and adaxial surfaces of *I. cordifolia* and *I. spinosa* stomatal were almost equal. The highest value of the stomatal index was observed on the adaxial surface of *I. hochstetteri* (27.46%), while the lowest value was observed on the abaxial surface of *I. oblongifolia* (9.95%). Haworth et al (2010) showed that stomatal index responded differently to the fluctuations of atmospheric CO_2_ in some Cupressaceae conifers. A recent study on the genus *Mimosa* (Fabaceae) revealed that the stomatal index was affected by some environmental factors such as temperature and precipitation (Ayala-Ramos et al. 2024).

Turgor-driven guard cell movements rely on the wall properties of guard cells and the surrounding epidermal (including subsidiary) cells (Gray et al. 2020). Zeiger (1983) suggested that the inability of stomata to close could be due to either improper protoplast function or to abnormal cell wall properties. The leaves of the studied taxa exhibited great diversity in epidermal cell shapes. Many forms, such as tetragonal, pentagonal, hexagonal, polygonal, and irregular, were recorded in most of the studied taxa of *Indigofera* (Quesada 1997; Zhao et al. 2022). However, the polygonal cell shape was not recorded on the abaxial surface of *I. spinosa*. Likewise, the irregular cell shape was not recorded in the leaf surfaces of *I. coerulea* var. *coerulea* and *I. oblongifolia*. Anticlinal cell wall patterns on the leaf surfaces can be used to separate the studied taxa. They varied among the studied taxa from straight to slightly undulated. In addition, they were deeply undulated in *I. spinosa* and *I. subulata* var. *subulata*, straight to slightly arched in *I. oblongifolia*, and somewhat arched to arched on the abaxial surface of *I. coerulea*. These features were found to have taxonomic significance, offering complementary data to aid in identifying certain species (Albert and Sharma 2013).On the other hand, the uniformity of thickness of the anticlinal walls was very useful in distinguishing taxonomic characteristics. The smooth anticlinal walls were prevalent among the studied taxa. However, some species showed different forms of wall thickness, such as smooth to beaded thickness on both surfaces and stem of *I. sessiliflora*, *I. cordifolia*, *I. spiniflora*, both surfaces of *I. colutea* and *I. spinosa*, the abaxial surface of *I. hochstetteri*, and the stem of *I. argentea*, *I. trita*, *I. subulata* var. *subulata*, and *I. hochstetteri*. The wall thickness was smooth to ridged on both leaf surfaces of *I. argentea*., and the adaxial surface of *I. hochstetteri*; ridged to knobbed on both surfaces of *I. subulata* var. *subulata*; and irregularly thickened on both leaf surfaces of *I. sessiliflora*, the adaxial surface of *I.cordifolia*, the abaxial surface of *I. spiniflora* and the stem of *I. oblongifolia*. These results match the findings of Kadiri et al. (2019) for the family Lauraceae. Stace (1965a) cited that species inhabiting drier habitats often attain straight to curved epidermal walls. However, the epidermal cell morphogenesis and anticlinal wall shape depend on actin and cellulose microfibril concentration and other signalling proteins (Bidhendi et al. 2019).

The cuticle is a superficial non-living layer produced by epidermal cells to limit the transpirational water loss and to help plants survive harsh conditions. The permeability of the cuticle relies largely on its thickness, structure, chemical composition, crystallization forms, and relative humidity (Bargel et al.^2004^; Iqbal et al. 2020). The leaf cuticle surface helped us to distinguish both surfaces of *I. hochstetteri* and the adaxial surface of *I. sessiliflora*, *I. trita*, *I.spiniflora*, and *I. argentea* from other studied taxa. It was reticulate in both surfaces of *I. cordifolia*, *I. subulata* var. *subulata* and the abaxial surface of *I. argentea*; ruminate in the adaxial surface of *I. argentea*, *I. sessiliflora*, *I. spiniflora*, and *I. trita*, the abaxial surface of *I. spinosa*, and both surfaces of *I. hochstetteri*; rugulate to ruminate in the abaxial surface of *I. colutea* and the adaxial surface of *I. spinosa* and *I. oblongifolia*; and rugulate in the rest of the studied taxa.

SEM analysis revealed two shapes of the waxy layer on leaf epidermal cells: a smooth, waxy layer was found in the epidermal cells of *I. trita*. Meanwhile, a scale-like waxy coating (lattice-like extrusions) was observed in the epidermal cells of the rest of the studied taxa (Xiang et al. 2010). Based on the results of this study, we noticed that the formation of crystals is uncommon among the species. The bio-mineralization of calcium oxalate crystals is a genetically regulated process controlled by environmental stresses such as soil salinity and calcium concentration; these crystals play a variety of physiological roles (Khan et al. 2023). Raphides and solitary crystals were observed on the leaves of *I. arabica* and *I. trita* that were collected from arid habitats (Sinai Peninsula, and Gebel Elba, respectively). In arid conditions, calcium oxalate crystals proved to capture non-atmospheric carbon at night, and during the day, crystal degradation provides subsidiary carbon for photosynthetic assimilation (Tooulakou et al. 2016). Metcalfe and Chalk (1950) have noted the presence of papillate projections on the abaxial surface of *Indigofera*. In this study, we recorded the existence of papillate protrusions in only one taxon (*I. argentea*).Similarly, Quesada (1997) reported that the papillate projection existed only in one species (*I. suffruticosa*) among the seven examined species of *Indigofera*. The frequency of epidermal cells is also of diagnostic importance. It was the highest on the adaxial and abaxial surfaces of *I. oblongifolia*, with an average epidermal cell frequency of 196.80. At the same time, it was the lowest on the abaxial surface of *I. trita,* with an average epidermal cell frequency of 49.17. Likewise, the epidermal cells of stems of the studied *Indigofera* exhibited a significant variation among the studied taxa. The most common shapes were tetragonal, pentagonal, hexagonal, polygonal, and elongated with straight to arched or slightly undulate anticlinal wall outlines. Polygonal cell shapes were present in all the studied taxa except *I. articulata, I.oblongifolia*, *I. hochstetteri*, and *I. spinosa*, while irregular shapes were present in *I. argentea* and *I. colutea*.

## Conclusion

In this study, we described the quantitative and qualitative characteristics of stomatal and epidermal cells of leaves and stems of 13 taxa of the genus *Indigofera* in Egypt. We recognized 19 normal stomata complexes (with nine subtypes) and seven abnormal stomatal complexes (with 13 subtypes). The occurrence of different types of abnormal stomata allowed us to design an artificial key to differentiate the taxa under investigation. Most leaves had slightly sunken to sunken stomata, while stomata were positioned at the same level as epidermal cells of stems of most studied taxa. *Indigofera*'s foliar and stem epidermal anatomy provides valuable insights into the taxonomy and differentiation of closely related taxa within the genus. Identifying stomatal types, frequency, and index contributes to the existing knowledge base and serves as a baseline for future research.

## Data Availability

All data generated or analyzed during this study is included in this published article.
