# Correction to: Taxonomic implications of normal and abnormal stomatal complexes in *Indigofera* L. (Indigofereae, Faboideae, Fabaceae)

**DOI:** 10.1007/s00709-024-01974-7

**Published:** 2024-08-02

**Authors:** Mohamed O. Badry, Ahmed K. Osman, Mostafa Aboulela, Shereen Gafar, Iman H. Nour

**Affiliations:** 1 Department of Botany and Microbiology, Faculty of Science, South Valley University, Qena, 83523 Egypt; 2 Department of Botany and Microbiology, Faculty of Science, Assiut University, Assiut, 71516 Egypt; 3 Department of Botany and Microbiology, Faculty of Science, Alexandria University, Alexandria, 21511 Egypt

**Correction to: Protoplasma**

10.1007/s00709-024-01951-0

There was a mistake in the date of collection of some species in Table 1 that samples 9, 10 and 13 were collected at the same date 25/3/2020 instead of 25/3/2006.

The Original article has been corrected.

